# Impact of aging and ergothioneine pre-treatment on naphthalene toxicity in lung

**DOI:** 10.1016/j.toxlet.2024.05.014

**Published:** 2024-05-18

**Authors:** Veneese JB Evans, Xiangmeng Wu, Kyle K Tran, Shanlea K Tabofunda, Liang Ding, Lei Yin, Patricia Edwards, Qing-Yu Zhang, Xinxin Ding, Laura S. Van Winkle

**Affiliations:** a Center for Health and the Environment, School of Veterinary Medicine, University of California-Davis, Davis, CA 95616-8732, USA; b Department of Anatomy, Physiology and Cell Biology, School of Veterinary Medicine, University of California-Davis, Davis, CA 95616-8732, USA; c Dept of Pharmacology and Toxicology, College of Pharmacy, University of Arizona, Tucson, AZ 85721-0207

**Keywords:** Naphthalene, Ergothioneine, Aging, Oxidative stress, Antioxidant, Pulmonary toxicity

## Abstract

Aging increases susceptibility to lung disease, but the topic is understudied, especially in relation to environmental exposures with the bulk of rodent studies using young adults. This study aims to define the pulmonary toxicity of naphthalene (NA) and the impacts of a dietary antioxidant, ergothioneine (ET), in the liver and lungs of middle-aged mice. NA causes a well-characterized pattern of conducting airway epithelial injury in the lung in young adult mice, but NA’s toxicity has not been characterized in middle-aged mice, aged 1–1.5 years. ET is a dietary antioxidant that is synthesized by bacteria and fungi. The ET transporter (ETT), SLC22A4, is upregulated in tissues that experience high levels of oxidative stress. In this study, middle-aged male and female C57BL/6 J mice, maintained on an ET-free synthetic diet from conception, were gavaged with 70 mg/kg of ET for five consecutive days. On day 8, the mice were exposed to a single intraperitoneal NA dose of 50, 100, 150, or 200 mg/kg. At 24 hours post NA injection samples were collected and analyzed for ET concentration and reduced (GSH) and oxidized glutathione (GSSG) concentrations. Histopathology, morphometry, and gene expression were examined. Histopathology of mice exposed to 100 mg/kg of NA suggests reduction in toxicity in the terminal airways of both male (p ≤ 0.001) and female (p ≤ 0.05) middle-aged mice by the ET pretreatment. Our findings in this study are the first to document the toxicity of NA in middle-aged mice and show some efficacy of ET in reducing NA toxicity.

## 1. Introduction

As humans transition from middle age to geriatric, 65 years and older (Colby and Ortman, 2014), they become more susceptible to xenobiotics found in the environment. Factors that may contribute to this susceptibility include medical and exposure history, declining immune responses, and reduced protection by antioxidants; all resulting in an increased risk of disease (Smithard and Yoshimatsu, 2022). Lung disease can be both exacerbated and initiated by environmental exposures (Shukla et al., 2018). Naphthalene (NA) is an abundant chemical, found in wildfire smoke, vehicle exhaust and tobacco smoke as well as a water contaminant from jet fuel (Risher et al. 2017) or in old coal and oil gasification sites (Turney and Goerlitz, 1990). NA has a well described injury and repair pattern in young adult mice, and this has been used to understand the mechanisms of Club cell replacement in distal conducting airways (Van Winkle et al. 1995; Van Winkle et al. 2002). However, NA injury and repair in aging lungs has not been previously investigated.

NA, abundant in urban areas, is metabolized by cytochrome P450 monooxygenase enzymes resulting in increased oxidative stress and cytotoxicity from reactive metabolites, e.g., naphthalene epoxide, 1,2-naphthoquinone (1,2-NQ) and 1,4-naphthoquinone (1,4-NQ). In the mouse lung, the conducting airways contain nonciliated bronchiolar epithelial Club cells which have abundant cytochrome P450 enzymes (CYP) and metabolize toxicants such as NA (Phimister et al. 2005b). NA targets Club cells in the lung regardless of the route of exposure (Plopper et al. 1992; Van Winkle et al. 1995). Typical routes of exposure to NA include inhalation and ingestion. The lung cellular target regardless of route of exposure is the nonciliated bronchiolar epithelial Club cell (Buckpitt et al. 2002; M. Abdo et al., 2001; Plopper et al. 1992; Shopp et al. 1984). NA causes oxidative stress-related damage to the conducting airway epithelium of the lung in both sexes with a slight sex difference in susceptibility in young adults; females are more susceptible (Carratt et al. 2016; Stelck et al. 2005; Van Winkle et al. 2002). However, the impact of NA on a middle-aged lung (approximately 1 year older) in either sex has not yet been investigated.

Endogenous antioxidants are important in protecting the lungs from oxidative stress. Glutathione (GSH) is key for NA detoxification as demonstrated in a series of studies using GSH depletion and/or blocking its repletion (Phimister et al. 2005a, 2005b; West et al. 2000; West et al. 2001a). GSH, the reduced form, is oxidized by glutathione peroxidase (GPx) to generate GSSG, which is reduced to GSH by glutathione S reductase (GSR). When in the reduced form, GSH can detoxify reactive NA metabolites creating NA-GSH conjugates, a process assisted by glutathione S transferase (GST) (Fanucchi et al. 2000; Forman and Zhang, 2021). The production of intracellular GSH is determined by glutamate cysteine ligase (GCL), which has two subunits: the modifier (GCLM) and catalytic (GCLC) subunits. The GCL enzyme is essential to GSH homeostasis inside the cell as it catalyzes the rate-limiting step in GSH synthesis- and adaptive upregulation of GSH levels have been attributed to increases in the expression of this enzyme (Orellana et al. 2017). A decline in GSH levels due to aging has been seen in rodent models as well as humans (Lu, 2009; Polonikov, 2020; Wang et al. 2003; Wu et al. 2004; Zhu et al., 2006). Thus, the process of aging can result in increased susceptibility to xenobiotic toxicants (Kurutas, 2016; Lange et al. 2012). Understanding the effects of oxidative stress on the middle-aged population requires knowledge of antioxidant levels in the context of aging.

Human studies have shown that ergothioneine (ET), a dietary antioxidant, protects against oxidant stress in the aging brain (Cheah et al. 2016a). ET is derived from bacteria and fungi, with the highest concentrations found in selective species of mushrooms, e.g. king bolete, and oyster mushrooms (Ey et al. 2007). ET, which is obtained solely through diet in humans and other animals, accumulates in tissues exposed to high levels of oxidative stress (Cheah and Halliwell, 2020; Cheah and Halliwell, 2021; Cheah et al. 2016b; Halliwell et al. 2018; Tang et al. 2018). There is still much to learn about the physiological function of ET, especially in the lung, but it has been studied in other organs, e.g., skin, heart, liver, and kidney (Arduini et al. 1990; Deiana et al. 2004; Liu et al. 2023; Salama and Omar, 2021). ET has a specific transporter (OCTN1 or ETT) encoded by the gene SLC22A4, which is upregulated in tissues that experience high levels of oxidative stress (Cheah et al. 2016a; Cheah and Halliwell, 2021; Halliwell et al. 2018).

Given the impact of NA in older, middle aged/aging mice is unknown, and the role of oxidant/antioxidant balance in epithelial biology of the aging lung is also unclear, our goal in the current study was to evaluate 1) basal antioxidant enzyme expression in aging mice, 2) responses of aging mice to NA exposure, and 3) susceptibility of the aging lung to NA-induced cytotoxicity when pretreated with ET.

## 2. Methods

### 2.1. Chemical sources

Naphthalene (NA) was purchased from Fisher Scientific (CAS-91–20-3) and was diluted in Mazola corn oil (vehicle control for NA treatment). Ergothioneine (ET) was purchased from BLDpharm (CAS-497–30–3) and was diluted in saline (vehicle control for ET exposures). Araldite 502 epoxy resin, dodecenyl succinic anhydride, and 2,4,6-Tris-(dimethylaminomethyl)phenol (DMP-30) were purchased from Ted Pella (Redding, CA). All other chemicals were reagent grade or better.

### 2.2. Animals

C57BL/6 J mice were purchased from Envigo and used to create a breeding colony. Mice were bred and housed at UC Davis on a scheduled 12-hour light/dark cycle from birth up to 1–1.5 years of age, which according to the Jackson Laboratory is equivalent to 42–56 years in humans, also referred to as middle aged (Flurkey et al. 2007). Pregnant dams and offspring were placed on an ET-free synthetic diet (AIN-93 G, Research Diets), which resulted in undetectable basal levels of ET in mice by HPLC-MS (< 0.4 μg/g), for the duration of the experiment. This defined diet was selected because it promotes normal mouse growth and is suitable for long term studies. Prior to NA treatment, aging mice were placed in groups of 4–6 and were given a unique access number for tracking and randomization/blinding purposes. All animals were euthanized with a lethal dose of pentobarbital i.p. Sentinel mice were housed in the same facility and tested negative for respiratory virus for the duration of the study. All animal experiments were performed under protocols approved by the University of California Davis IACUC in accordance with National Institutes of Health guidelines.

### 2.3. Experimental design

**Fig. 1.**
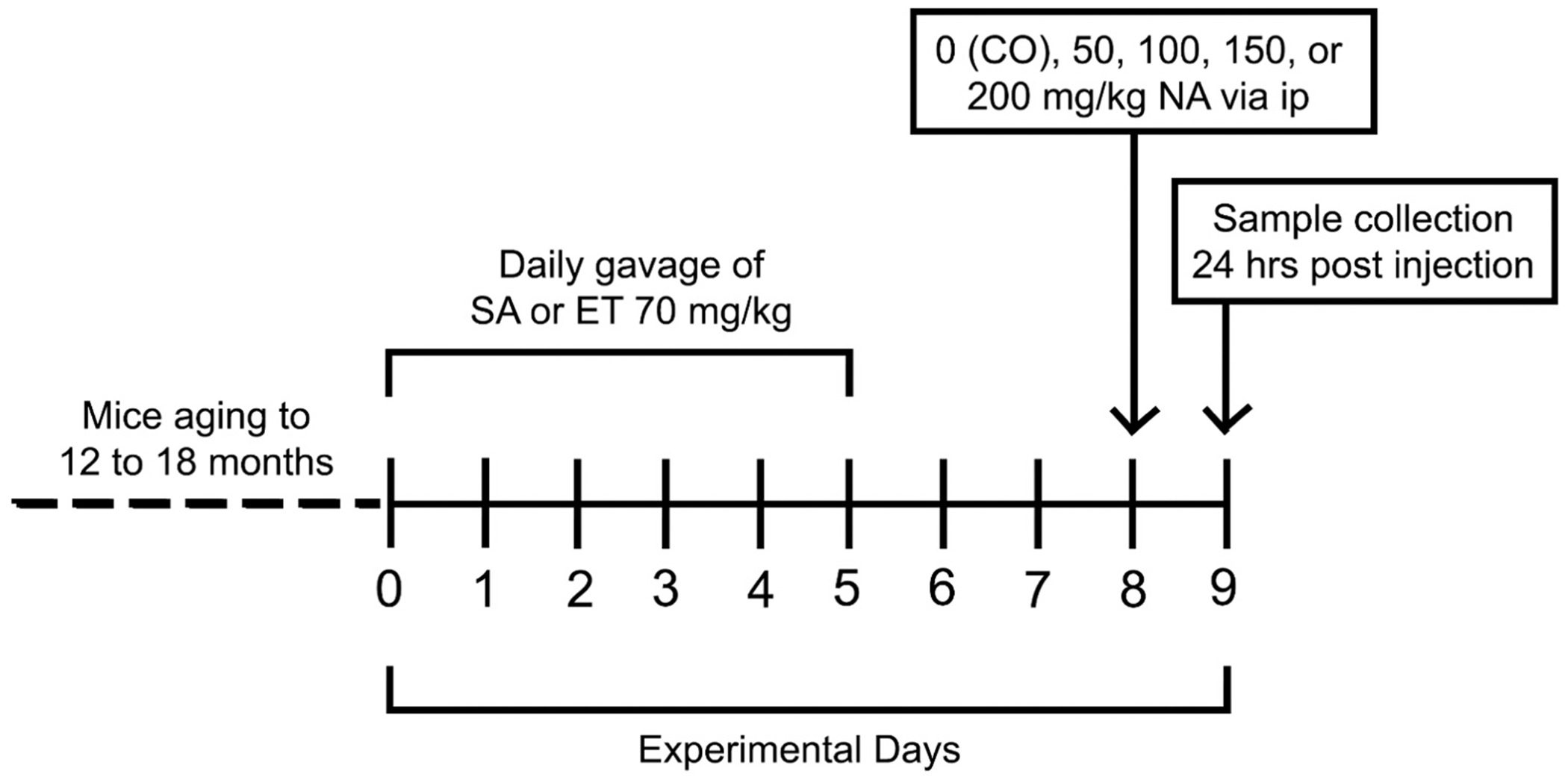
Experimental design. TExperimental design with mice aging to 12–18 months while on a defined diet. Abbreviations: SA, saline; CO, corn oil; ET, ergothioneine; NA, naphthalene.

Mice from different breeding pairs were randomly placed into experimental groups. Groups (n=5) were either treated with saline (SA) or 70 mg/kg of ET by gavage for 5 days (Fig. 1 – Experimental design). This dose of ET was selected because it was the highest dosage that was used in prior studies and has been shown to be both safe and effective in mice (Tang et al. 2018). Mice were then, two days after the last ET gavage, given corn oil (CO) or NA at 50 mg/kg, 100 mg/kg,150 mg/kg, and 200 mg/kg ip. Doses were selected from prior studies in juvenile and adult mice to facilitate comparisons with prior work and future work with mice of different ages where respiratory rate and body size would complicate dose delivered equivalencies if the compound was inhaled (Carratt et al. 2019a; Fanucchi et al. 1997; Plopper et al. 2001; Van Winkle et al. 1995; West et al. 2001b). We elected to use ip injection because this allows control of dose delivered and facilitates studies of temporal responses in the Club cell injured population, which is the cellular target regardless of route. At 24 hours post injection, the lungs were extracted, and the left lung was stored inflated at 30 cm of pressure in Karnovsky’s fixative for embedment in araldite resin for high resolution light microscopy and stereology (Carratt et al. 2019b; Plopper et al. 1991; Sutherland et al. 2010; Van Winkle et al. 1996). The right lung from the same animal was used for RNA preparation for qRT PCR after inflation with RNALater. The time point of 24 hours post NA exposure enables observation of maximal cytotoxicity at the lung epithelium, as has been demonstrated in prior studies (West et al. 2002).

### 2.4. Lung microdissection and real-time PCR

The right lung lobe was cannulated at the trachea and fully inflated with RNA-later (Ambion/Applied Biosystems; Foster City, CA). The liver was diced into small cubes and stored in RNA-later. All samples were stored at −20 °C. RNA was isolated from homogenized lungs and microdissected into three lung sub compartments: proximal airway, distal airway and parenchymal tissue (Baker et al. 2004). The proximal airway included the intrapulmonary bronchi and larger intrapulmonary bronchioles; the distal airway included the smaller bronchioles and terminal airways; and the parenchyma was primarily composed of connective tissues and alveoli. RNA was isolated using a RNeasy Plus Mini kit with a gDNA elimination column (Qiagen; Hilden, Germany), and quantified with a NanoDrop spectrophotometer (ThermoFisher; Waltham, MA). The real-time PCR system (StepOnePlus; Applied Biosystems) was utilized to analyze the following genes of interest using Taqman assays (ThermoFisher; Waltham, MA) shown in Table 1. Genes were normalized to the housekeeper, RPl13a, and then to the male parenchyma of the SA/CO or CO-only group. The rationale for normalizing to the males rather than females is because it is known that females are more susceptible to NA toxicity compared to males (Van Winkle et al. 2002). Out of the 3 lung regions we chose to normalize to the parenchyma region due to the absence of airways, compared to the proximal and distal conducting airways; further, this facilitates direct comparison of male and female responses to each other.

### 2.5. Resin embedment and high-resolution light microscopy

The left lobes of the mice were fixed at 30 cm of constant pressure, stored in Karnovsky’s fixative, and cut into 4 separate pieces, exposing the airways. The samples were processed in Zetterquist’s buffer and embedded into Araldite 502 epoxy resin. Two of the four lung samples were randomly selected for sectioning at 1 μm on a microtome with glass knives. The sectioned tissue was then placed on a gelatin slide, stained with methylene blue/Azure II, and imaged on a high-resolution bright field Olympus BH-2 microscope at 20x magnification.

### 2.6. Stereology

Lung tissue sections were imaged by a high-resolution brightfield microscope for stereological assessment of the epithelium in the conducting airways using standard morphometric approaches (Hsia CCW et al., 2010; Murphy et al. 2013). The mass (Vs or Volume per surface) of the epithelium was measured using a counting system of points (P) and intercepts (I) on a cycloid grid in Stereology toolbox software (Kelty et al. 2020). Volume fraction was calculated using the following formula: Vs= (l/p)(ΣPts/ΣInt)(1/2), where Vs is the mass (μm^3^/μm^2^), (l/p) is the length and sum of curves divided by the sum of points, and (ΣPts/ΣInt) is the sum of points of either all, vacuolated, or non-vacuolated conducting airway epithelial cells divided by the sum of the intercepts of the basal lamina of the epithelium. The results of (ΣPts/ΣInt) is represented as thickness or ͳ_epi_. The volume fraction (percentage Vv) was calculated using the equation: Vv= ΣPts / P_t_, where the sum of vacuolated points, ΣPts, is divided by the total cells counted, P_t_, which includes both vacuolated and non-vacuolated cells.

### 2.7. Mass spectrometry

Frozen blood, liver, and lung samples were collected 24 hours post NA exposure. Tissue samples were homogenized in 19x volumes (v/w, i.e. 1 mg liver or lung added to 19 μL buffer) of Tris-acetate buffer (100 mM Tris-base, 1.0 mM ethylenediaminetetraacetic acid (EDTA), 150 mM potassium chloride (KCl), pH=7.4) in an ice bath using a Polytron Bio-Gen Series PRO200 powered homogenizer (speed level 3 for 10 seconds).

Method for detection of GSH/GSSG was adopted from prior studies (Kelty et al. 2022). GSH/GSSG levels in liver and lung were determined by processing tissue homogenates using protein precipitation and liquid-liquid extraction as follows: 20 μL internal standard, GSH-^13^C2,^15^N (6 μL/mL in 10 % acetonitrile; Sigma Aldrich), was added to 50 μL tissue homogenate to account for any losses in sample processing and followed by the addition of 50 μL 1 N HCl. The samples were vortexed for 10 s at room temperature (RT) and centrifuged in an Eppendorf 5424 R centrifuge at 14,000 rpm, 4 °C, for 10 min. Then 50 μL supernatant was transferred to a new 1.5-mL plastic tube and combined with 350 μL water and 500 μL dichloromethane, vortexed for 30 s at RT and centrifuged at 4 °C and 14,000 rpm for 10 min. The supernatant was collected and centrifuged a second time; 5 μL of the resultant supernatant was injected into the LC-MS/MS for GSH/GSSG quantification.

Method for detection of ET was modified from (Cheah et al. 2016a; Cheah et al. 2016b). To analyze ET levels, 20 μL of blood or tissue homogenates were mixed with 10 μL internal standard (hercynine-d9, 5 μg/mL, Tetrahedron) and 150 μL methanol, and then vortexed for 10 s at room temperature and centrifuged in an Eppendorf 5424 R centrifuge at 14,000 rpm and 4 °C for 10 min. The supernatant was then diluted with 800 μL water and processed using IsoluteC18 25-mg/1-mL solid phase extraction (SPE) cartridges (Biotage, Charlotte, North Carolina). The column was activated with 1 mL methanol, equilibrium with 1 mL water, then the diluted sample was loaded, followed by collection of the unbound fraction. The collected fraction was then centrifuged at 14,000 rpm for 10 min at 4 °C and 2 μL of the resultant supernatant was injected into the LC-MS/MS for ET quantification. The LC-MS system consisted of an Agilent model 1290 HPLC (Agilent, Santa Clara, California), and a Qtrap 6500 plus mass spectrometer (Sciex, Ontario, Canada) equipped with a Turbo IonSpray source. Analyst Software 1.6.3 was used for data acquisition and processing.

### 2.8. Statistics

Goodness of fit testing was conducted on all data sets using Shapiro-Wilk test in ProUCL 5.2. Normality testing was also conducted in GraphPad Prism for comparison. Differences between age, sex, and/or treatment groups were evaluated using two-way analysis of variance (ANOVA) for normally distributed data sets in Graphpad Prism. Significance found between groups was based on Tukey’s multiple comparisons test (Tukey’s HSD) with significance set at P< 0.05. Data sets that were not normally distributed were analyzed using the non-parametric Kruskal-Wallis test with significance set at P< 0.05. All datasets were analyzed for outliers through the Grubbs’ Test, resulting in significant outliers (p-value = 0.05) being omitted. Error bars are presented using standard error of mean, or SEM.

## 3. Results

### 3.1. Gene expression in middle-aged sham treated lung

**Fig. 2.**
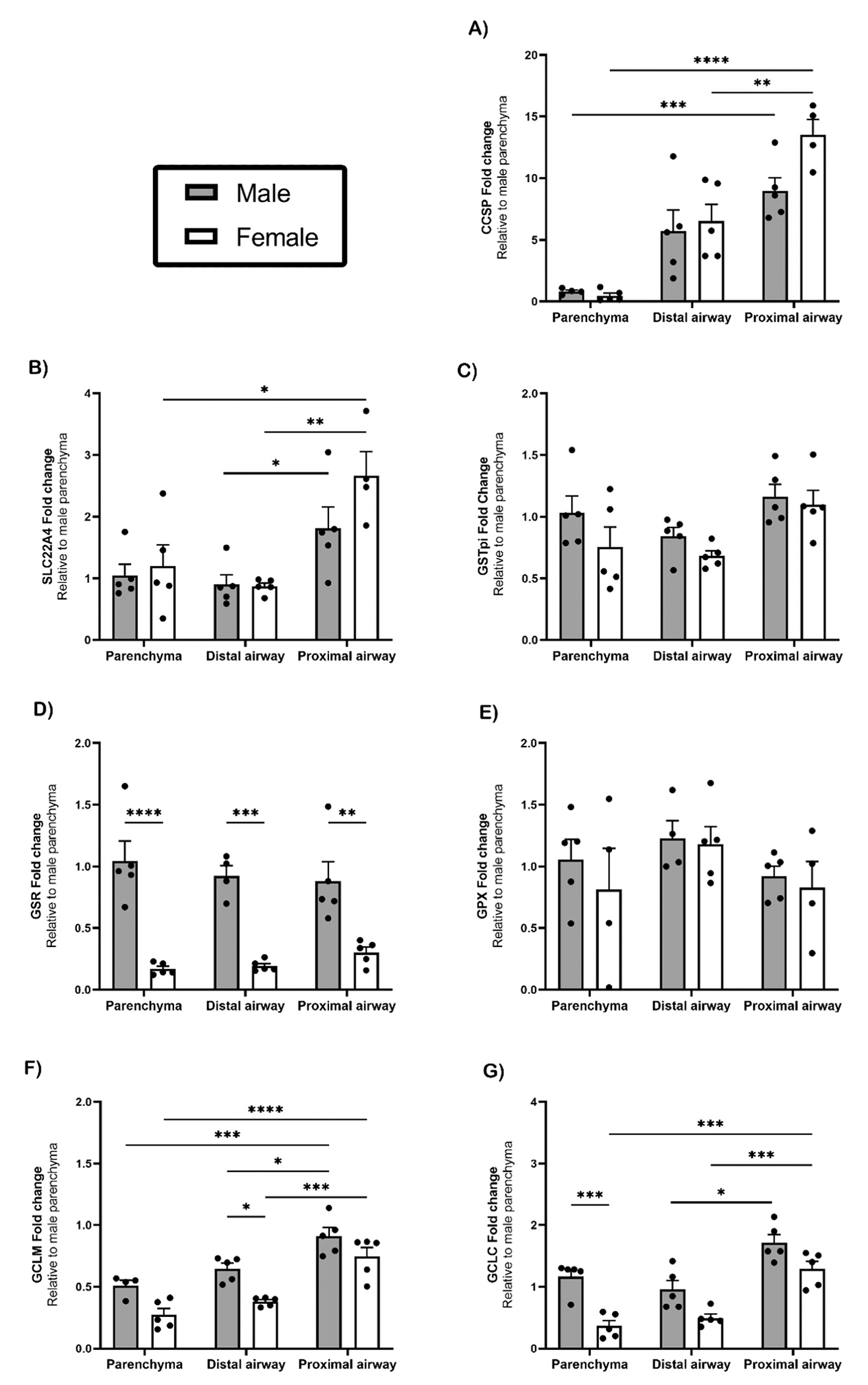
Site and sex specific differential expression of genes in the lung. Gene expression was determined by qRT-PCR in relation to Rpl13a as a housekeeping gene. CCSP [A], SLC22A4 [B], GSTpi [C], GSR [D], GPx [E], GCLM [F] and GCLC [G] were measured in lung samples from untreated male and female middle-aged mice (1–1.5 years) n= 4–5. Statistical analysis by 2-way ANOVA for normally distributed data sets, and non-parametric Kruskal-Wallis test for datasets not normally distributed. *, p ≤ 0.05; **, p ≤ 0.01; ***, p ≤ 0.001; ****, p ≤ 0.0001. Abbreviations: SLC22A4, ergothioneine transporter; GSTpi, glutathione S transferase; GSR, glutathione S reductase; GPx, glutathione peroxidase; GCLM, glutamate-cysteine ligase modifier subunit; GCLC, glutamate-cysteine ligase catalytic subunit.

Gene expression in three microdissected regions from male and female lungs was analyzed for key elements of phase two metabolism involving conjugation and detoxification, as well as other cellular markers, with fold change relative to middle-aged male parenchyma. Club cell secretory protein (CCSP) was expressed at significantly greater levels (p ≤ 0.01) in the female proximal airway compared to the distal airway (Fig. 2A). In addition, there was a significantly greater expression of CCSP in the male (p ≤ 0.001) and female (p ≤ 0.0001) proximal airway compared to the parenchyma (Fig. 2A). Similarly, to CCSP expression, ergothioneine transporters (SLC22A4) in middle-aged males (p ≤ 0.05) and females (p ≤ 0.01) were significantly greater in the proximal airway compared to the distal airway (Fig. 2B). There was no significant difference in regard to sex or location for expression of glutathione transferase (GSTpi) (Fig 2C). Glutathione reductase (GSR) in the parenchyma (p ≤ 0.0001), distal airway (p ≤ 0.001), and proximal airway (p ≤ 0.01) was significantly lower in females compared to males (Fig. 2D). In contrast, glutathione peroxidase (GPx) showed no significant difference in regard to sex or location (Fig. 2E). Glutamate cysteine ligase modulator subunit (GCLM) (Fig. 2F), and catalytic (GCLC) (Fig. 2G) was significantly expressed in the proximal airway compared to the distal airway, having the greatest expression in males compared to females in all lung regions (p ≤ 0.001).

### 3.2. Impact of aging on genes related to the abundance of oxidized and reduced GSH in lung tissue

**Fig. 3.**
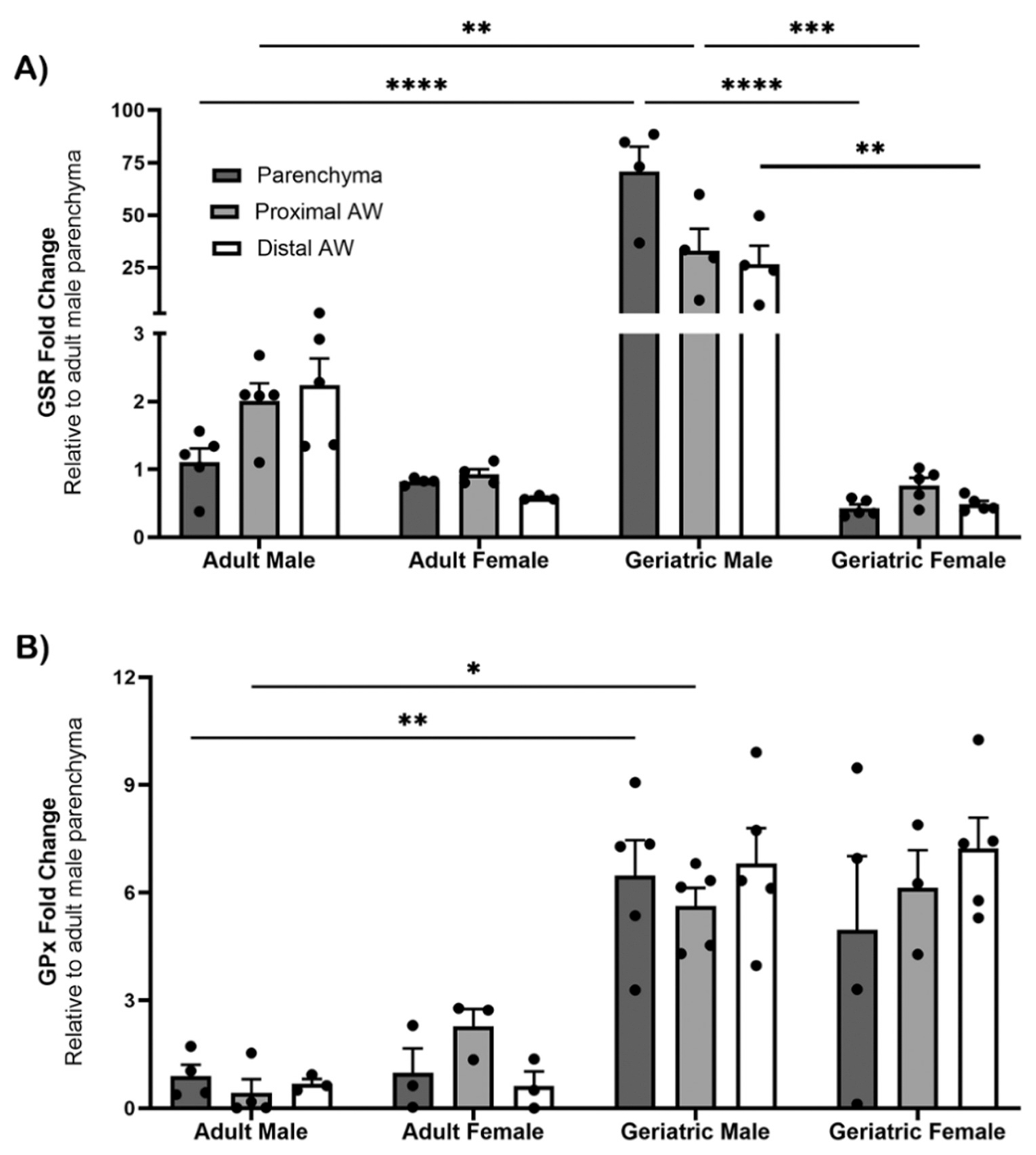
Comparison of gene expression of GSR and GPx in the microdissected lung. The lungs of male and female adult (2–3 months) and middle-aged (1–1.5 years) mice in the control group were microdissected and analyzed for GSR [A] and GPx [B] expression. Gene expression was determined by qRT-PCR in relation to Rpl13a as a housekeeping gene. Values are standard error of the mean fold change normalized to the adult male parenchyma region (n= 3–5). Statistical analysis by 2-way ANOVA for normally distributed data sets, and non-parametric Kruskal-Wallis test for datasets not normally distributed. *, p ≤ 0.05; **, p ≤ 0.01; ***, p ≤ 0.001; ****, p ≤ 0.0001. Abbreviations: AW, Airway; GSR, glutathione S reductase; GPx, glutathione peroxidase.

To determine whether the reduction in GSR levels in middle-aged female proximal and distal airways, compared to males, was due to aging, we compared the adult and middle-aged control groups for gene expression of GSR and GPx, with fold change relative to adult male parenchyma (Fig. 3). We found that expression of GSR in the proximal and distal airways of adult mice was not significantly different by sex, unlike in the middle-aged mice. Middle-aged males had greater expression of GSR than adult males in the parenchyma (p ≤ 0.0001), and proximal (p ≤ 0.01) airways (Fig. 3A). Expression of GSR was significantly lower in the parenchyma (p ≤ 0.0001), proximal (p ≤ 0.001), and distal airways (p ≤ 0.01) of middle-aged females than males (Fig. 3A). Strikingly, GPx was highly expressed in middle-aged mice compared to adults, in all lung regions in both males and females (Fig. 3B).

### 3.3. ET levels in whole lung, liver, and whole blood

**Fig. 4.**
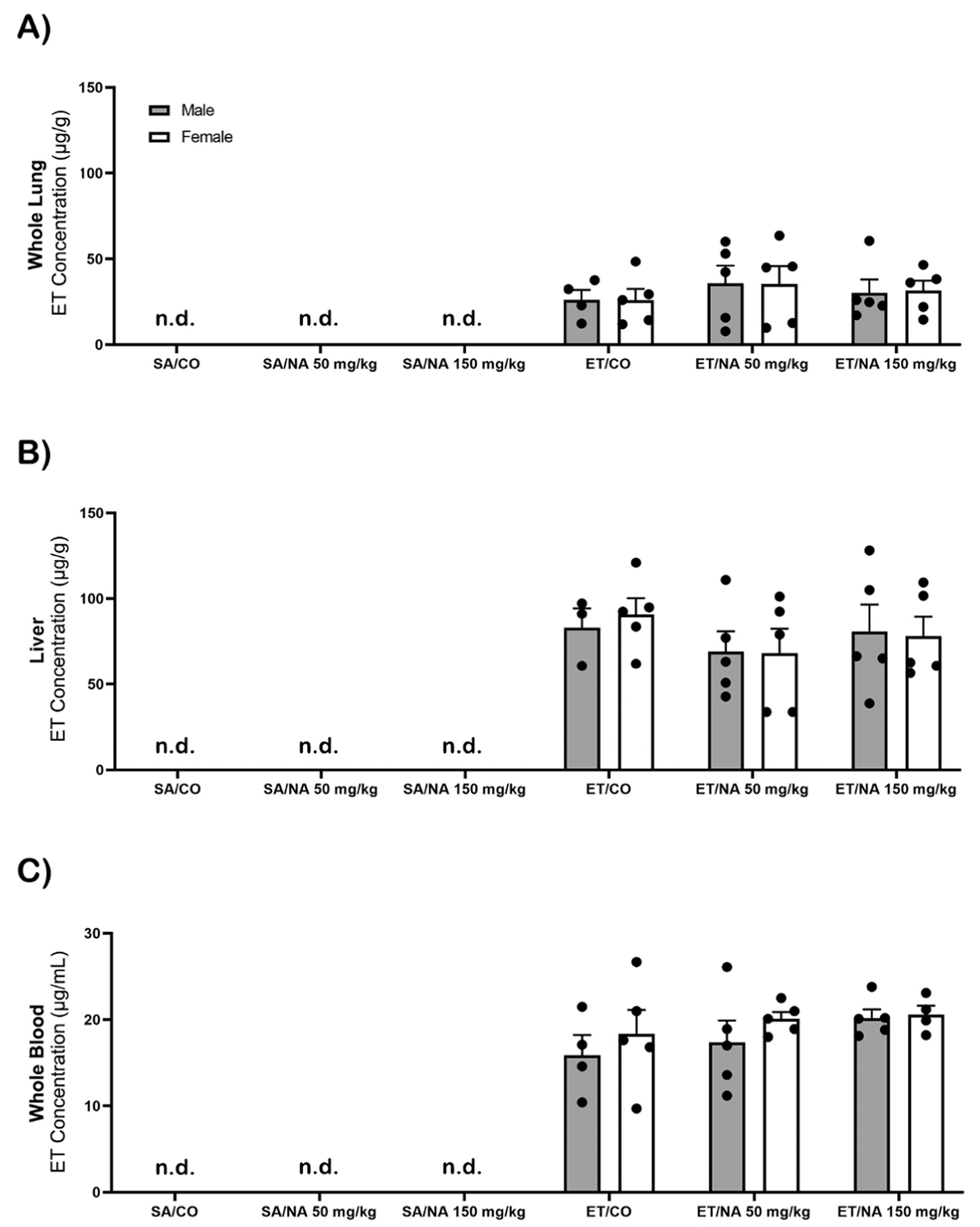
Accumulation of ET & lack of ET concentration changes in whole lung, liver, and whole blood after NA exposure. Middle-aged mice (n=4–5) were treated with 70 mg/kg ET for 5 consecutive days. The lung [A], liver [B], and blood [C] were analyzed for ET concentration 24 hours post exposure to 150 mg/kg of NA using HPLC-MS. The limit of detection was 0.02 μg/mL in blood and 0.4 μg/g in tissue samples. Two-way ANOVA showed no significance between male and females in the treated group. Abbreviations: n.d., not detected; SA, saline; CO, corn oil; ET, ergothioneine; NA, naphthalene.

Middle-aged mice treated with SA (n= 4–5) and maintained on an ET free diet had undetectable levels of ET in lung (Fig. 4A), liver (Fig. 4B), and blood (Fig. 4C). The minimum detectable level of ET in blood was 0.02 μg/mL, and 0.4 μg/g in tissue samples. All samples collected from mice treated with ET had detectable levels of ET. Liver had greater concentration of ET compared to lung. There was no significant difference between sex or exposure groups in the blood and tissue samples.

### 3.4. GSH, GSSG and GSH/GSSG in the lung and liver

To assess the capacity of the prevalent endogenous antioxidant GSH to mitigate oxidative stress in middle-aged mice, we measured the concentration of oxidized and reduced glutathione in both the lung and liver using HPLC-MS. In the lung, there was no significant difference in the GSH concentration in both male and female mice between saline and ET treated groups or among groups treated with 0, 50, and 150 mg/kg of NA, at 24 hours after exposure (Fig. S1A, S1B). When male mice were treated with ET and exposed to 50 mg/kg of NA, the GSSG level in the lungs increased by 2-fold (p ≤ 0.01) compared to the control. Additionally, ET treated mice had a 2-fold greater (p ≤0.01) concentration of GSSG at 50 mg/kg than untreated mice. As the concentration increased from 50 mg/kg to 150 mg/kg of NA the GSSG concentration significantly decreased by 2-fold in the ET treated male group (Fig. S1C). As for females, there was no significant difference in GSSG concentration between the treated and untreated groups (Fig. S1D). The GSH/GSSG ratio, an indicator of cellular health, had no significant differences between the groups of male and female mice (Fig.S1E, S1F).

**Fig. 5.**
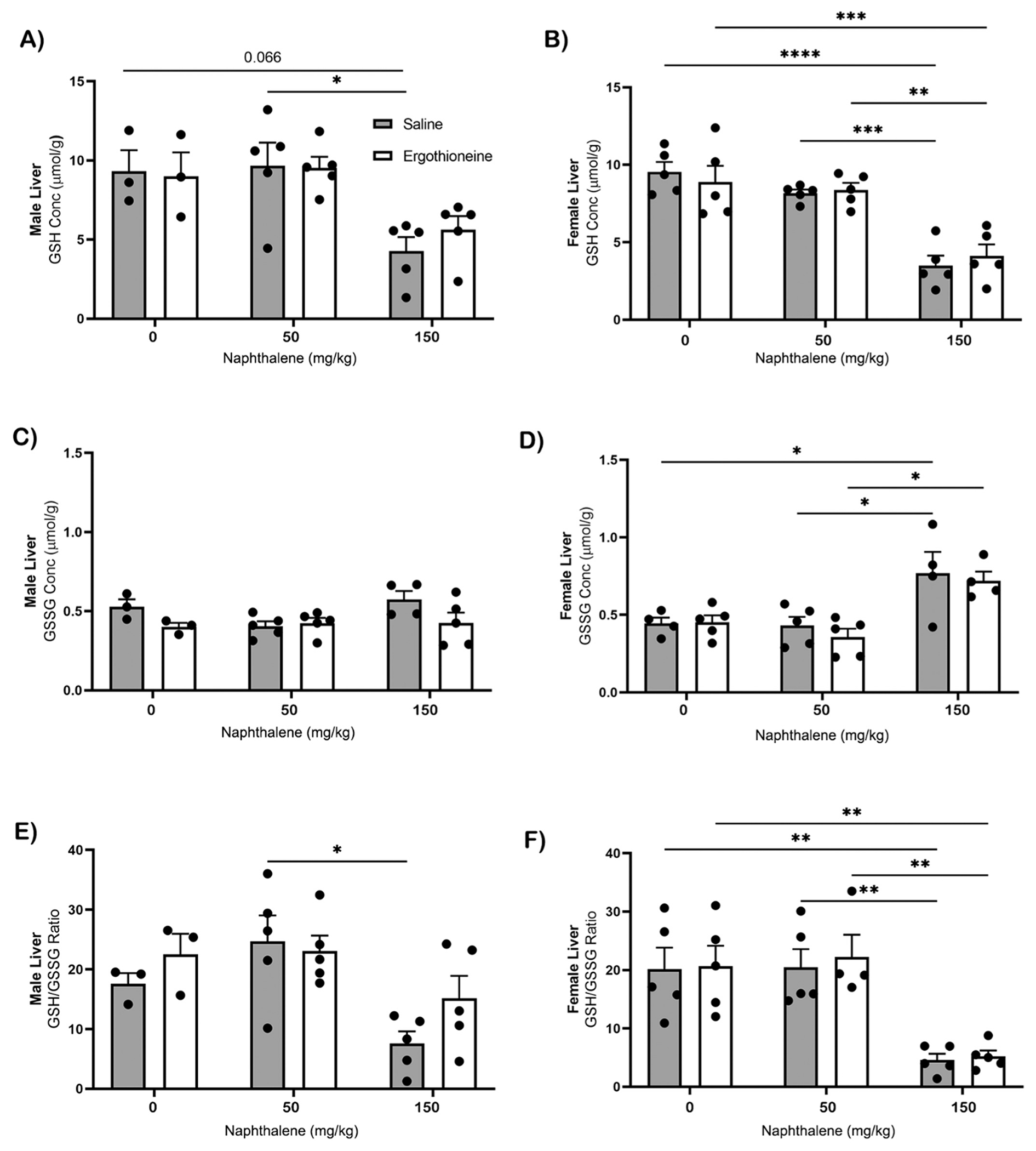
GSH, GSSG and GSH/GSSG ratio in the liver. Male and female middle-aged mice (n=3–5) have GSH [A-B], GSSG [C-D] levels measured using HPLC-MS post ET pretreatment and 24 hours after NA exposure to 50 and 150 mg/ kg i.p. GSH/GSSG ratio, an indicator of liver health, was also evaluated in both males [E] and females [F]. Statistical analysis by 2-way ANOVA. *, p ≤ 0.05; **, p ≤ 0.01; ***, p ≤ 0.001; ****, p ≤ 0.0001. Abbreviations: ET, ergothioneine; NA, naphthalene.

GSH concentrations in the liver decreased by 2-fold in both males (p ≤ 0.05) and females (p ≤ 0.001) as NA concentration increased (Figs. 5A, 5B). ET treated female mice had a 2-fold decrease (p ≤ 0.01) in GSH as NA concentration increased (Fig. 5B). GSSG in the liver showed no significant difference in males (Fig. 5C). Female GSSG levels significantly increased in both the ET treated and untreated groups (p ≤ 0.05) as the NA concentration increased (Fig. 5D). Male and female GSH/GSSG ratio in the liver of the untreated group significantly decreased 2-fold (p ≤ 0.05) from 50 mg/kg NA to 150 mg/kg NA. This was also observed in ET treated mice, however the ET treated male liver exposed to 150 mg/kg NA had greater ratios than the saline treated mice exposed to 150 mg/kg, although not significant (Figs. 5E, 5F).

### 3.5. Gene expression related to GSH production and detoxification pathways in lung tissue

To establish if a NA exposure at various doses impacts GCLM and GCLC, we measured gene expression in both microdissected lung (Fig. S2) and whole liver (Fig. S3A, S3B) in middle-aged male and female mice. Expression of GCLM and GCLC significantly increased at the maximum NA exposure dose of 200 mg/kg compared to 0 mg/kg, 50 mg/kg, 100 mg/kg, and 150 mg/kg in both the proximal (p ≤ 0.0001) and distal airway (p ≤ 0.05) (Fig. S2). There was no significant difference of GCLM and GCLC expression in the male liver after NA exposure. In the female liver, GCLM was lower at 200 mg/kg compared to 0 mg/kg (p = 0.07) and 100 mg/kg (p ≤ 0.05) of NA (Fig. S3A). GCLC gene expression in the female liver was significantly lower at 150 mg/kg (p ≤ 0.05) and 200 mg/kg (p ≤ 0.01) compared to 100 mg/kg NA (Fig. S3B).

**Fig. 6.**
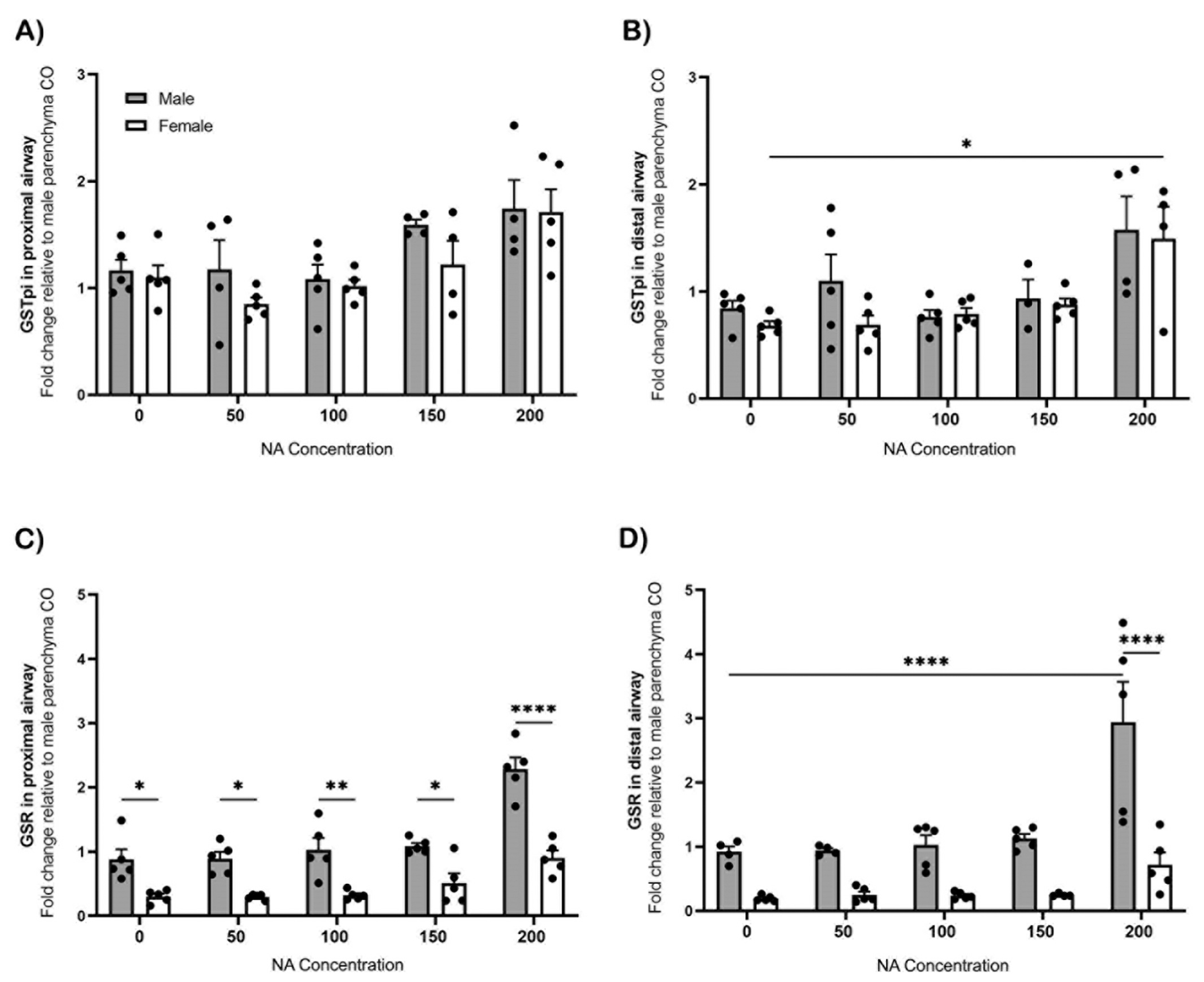
Gene expression related to GSH detoxification in NA exposed microdissected lungs. Microdissected lung samples were collected from NA exposed male and female middle-aged mice (1–1.5 years). GSTpi [A,B] and GSR [C-D] gene expression was determined using qRT-PCR in relation to Rpl13a as a housekeeping gene. NA dose response included 0 mg/kg (CO), 50 mg/kg, 100 mg/kg, 150 mg/kg, and 200 mg/kg. Values are standard error of the mean fold change normalized to the male parenchyma region of the CO group (n= 4–5). Statistical analysis by 2-way ANOVA. *, p ≤ 0.05; **, p ≤ 0.01; ****, p ≤ 0.0001. Abbreviations: NA, naphthalene; CO, corn oil.

GSTpi in the male liver was significantly greater at 150 mg/kg (p ≤ 0.01) and 200 mg/kg (p ≤ 0.05) of NA compared to 0 mg/kg (Fig. S3D). When comparing the GSTpi gene expression between sexes, males have a significantly greater expression at 150 mg/kg (p ≤ 0.0001) and 200 mg/kg (p ≤ 0.0001) compared to females (Fig. S3D). GSTpi in the female proximal (Fig. 6A) and distal (Fig. 6B) airways, exposed to NA at various doses, had a significant increase (p ≤ 0.05) at the highest NA dose of 200 mg/kg when compared to 0 mg/kg NA.

GSR expression in the male liver had no significant difference post NA exposure (Fig. S3C). The GSR expression in female liver was significantly lower at 200 mg/kg (p ≤ 0.05) of NA compared to 100 mg/kg (Fig. S3C). GSR in the proximal (Fig. 6C) and distal airway (Fig. 6D) both had a significant sex difference (p ≤ 0.05) at each NA dose, with males having the greatest expression of GSR at 200 mg/kg compared to females.

CCSP expression was also measured in the proximal (Fig. S4A) and distal (Fig.S4B) airways in middle-aged male and female mice. CCSP expression in both male and female proximal airway was significantly less (p ≤ 0.01) at 200 mg/kg compared to 0 mg/kg NA. Male proximal airway CCSP expression was significantly less (p ≤ 0.05) at 200 mg/kg compared to 50 mg/kg. CCSP expression in female distal airway was significantly less (p ≤ 0.01) at 150 mg/kg compared to 0 mg/kg NA.

### 3.6. Histology and stereology of ET pre-treated lungs post NA exposure

**Fig. 7.**
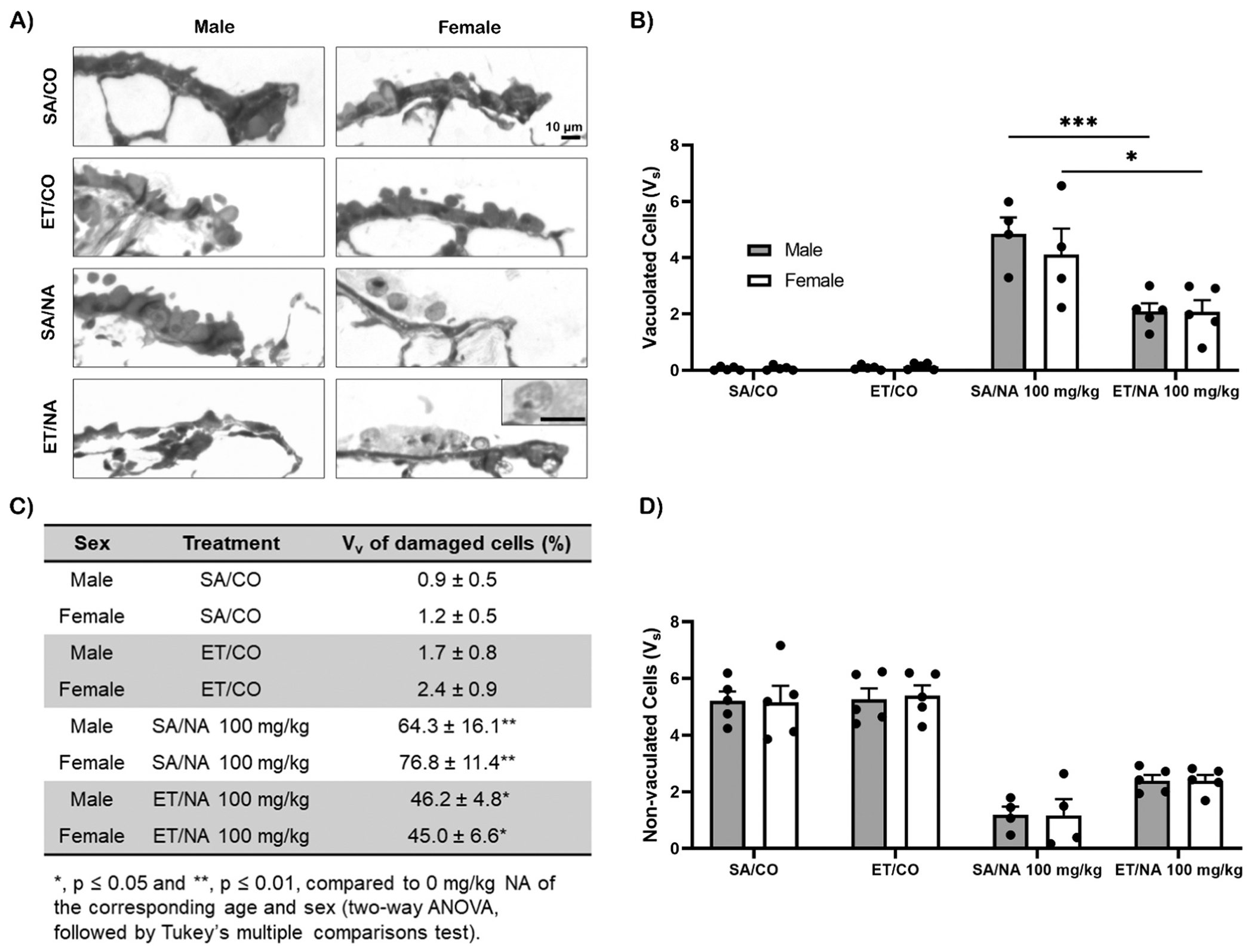
Histology and stereology of ET treated middle-aged terminal airway exposed to 100 mg/kg NA. Male and female middle-aged mice (n= 4–5) were treated with ET prior to a 24-hour exposure period to 100 mg/kg of NA ip. The airway and terminal airways were imaged using a high-resolution light microscope at 20x and 40x, then converted to grayscale [A]. Stereology was conducted using the imaged lungs and the vacuolated cells [B], non-vacuolated cells [D], and the volume fraction (Vv) of damaged cells (presented as mean ± SD) were calculated (n= 4–5) [C]. Error bars represent standard error. Statistical analysis by 2-way ANOVA. *, p ≤ 0.05; **, p ≤ 0.01; ***, p ≤ 0.001. Abbreviations: SA, saline; ET, ergothioneine; NA, naphthalene; CO, corn oil.

To understand the effect of ET pretreatment on NA epithelial toxicity, we examined the proximal and terminal airways using high resolution light microscopy in resin sections which allows for visualization of cytoplasmic vacuolization. When comparing the SA/CO group (control) to the ET/CO group in the larger proximal airway and distal terminal bronchioles, the ET treated shows no evidence of necrosis and looks similar to the control SA/CO. The effects of 100 mg/kg of NA in the larger proximal airway and distal terminal bronchioles of male and female mice were similar; both locations had necrosis, evident as swelling and vacuolation of airway epithelial Club cells. However, females had a greater distribution of cell vacuolation and some cells had already been exfoliated, indicating a more advanced stage of lung injury compared to males at the same dose (Figs. 7A, S5A). When we treated the mice with ET prior to the NA exposure, males had reduced Club cell necrosis in the proximal airway and terminal bronchioles compared to the mice not treated with ET. The proximal airway of the female in the ET pretreated group exposed to NA has far less vacuolated cells compared to the mice not pretreated with ET. There was no significant difference between the female ET treated and untreated exposed to NA in the terminal bronchiole (Figs. 7A, S5A). Overall, based on histology, middle-aged males treated with ET and exposed to NA contained fewer vacuolated cells than in females at 24 hrs post NA exposure.

Based on previous data from NA dose response for gene expression of CCSP (data not shown), which indicated a threshold for frank toxicity, we decided to conduct stereology on mice treated with ET and exposed to 100 mg/kg of NA due to the assumption that this dose is the lowest observed effect level in middle aged mice. Thus this dose was used to test the role of ET protection. Using stereology, we measured vacuolated cells, non-vacuolated cells, and total thickness of the lung epithelium in the proximal and terminal airways of ET treated mice exposed to 100 mg/kg NA. In the proximal airway, male and female mice in the control group (treated with SA, and exposed to CO), had significantly lower vacuolated cell mass (p ≤ 0.01) compared to the saline treated group exposed to 100 mg/kg NA (Fig. S7B). Female mice treated with ET and exposed to NA had significantly greater mass of vacuolated cells (p ≤ 0.05) compared to female mice treated with ET and exposed to CO (Fig. S5B). When measuring non-vacuolated cells, we observed opposing results from our vacuolated measurements. Male and female mice treated with SA and exposed to CO had significantly greater (p ≤ 0.001) non-vacuolated cells compared to mice treated with SA and exposed to NA (Fig. S5C). ET treated female mice exposed to CO has significantly greater (p ≤ 0.001) non-vacuolated cells compared to female mice treated with ET and exposed to NA (Fig. S5C). There was no significant difference in the lung epithelium total thickness and the V_v_ of damaged cells (data not shown).

The terminal airway had similar vacuolated cell pattern as the proximal airway, with the control group having significantly lower (p ≤ 0.0001) vacuolated cells compared to the saline treated mice exposed to 110 mg/kg NA (Fig. 7B). There was a significant treatment difference in vacuolated cells in both male and female terminal airways. Vacuolated cells were significantly lower in both male (p ≤ 0.001) and female (p ≤ 0.05) distal airways treated with ET compared to those that were treated with saline post NA exposure (Fig. 7B). The volume fraction of damaged cells, significant increased (p ≤ 0.05) in every group that was treated with NA when compared to the control groups of the corresponding age and sex (Fig. 7C). Non-vacuolated cells were significantly lower (p ≤ 0.0001) in groups exposed to NA (Fig. 7D).

### 3.7. ET transporter gene expression in NA treated lung and liver

**Fig. 8.**
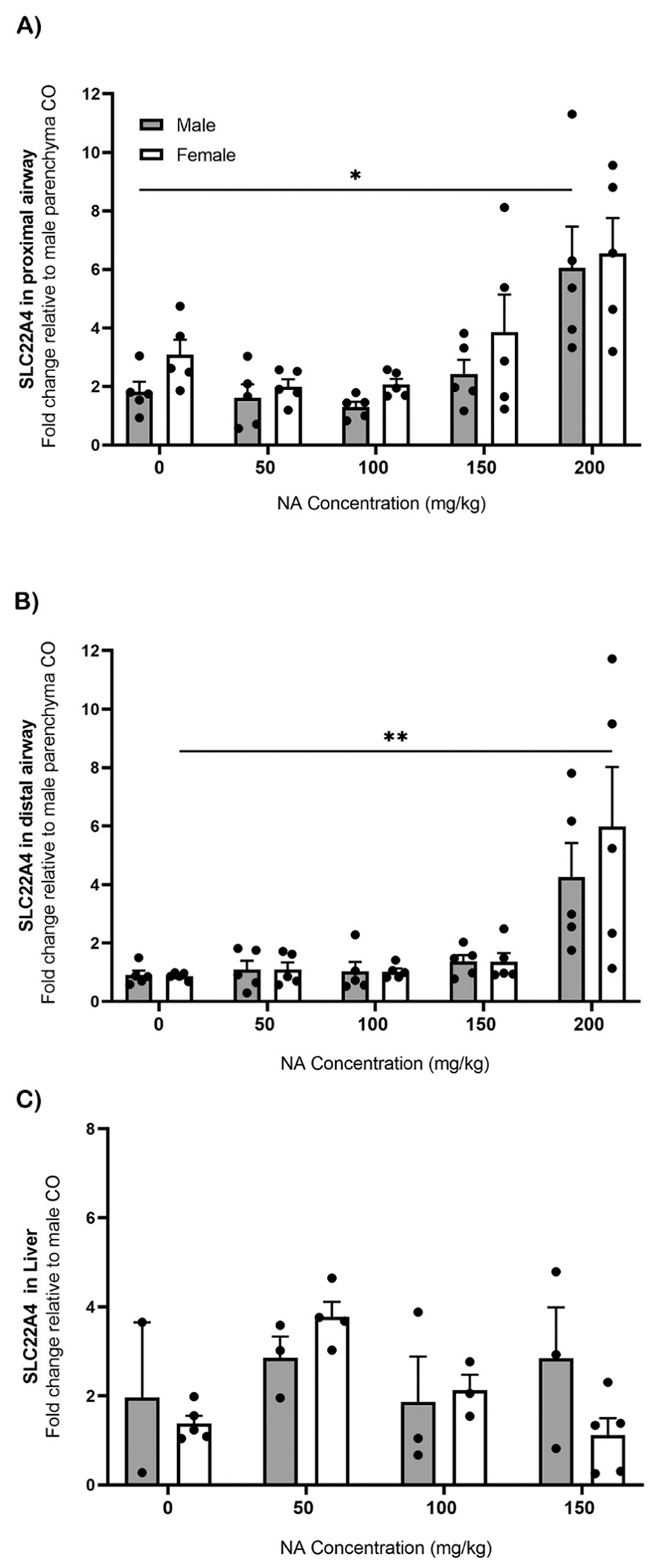
ET transporter changes in response to NA in middle-aged mice lung and liver. SLC22A4 (ET transporter) was measured in microdissected lung [A,B] and liver [C] samples from untreated NA exposed middle-aged mice (n=2–5) by qRT-PCR. Both the proximal [A] and distal [B] airways of the lungs were analyzed in a NA dose response of 0 mg/kg (CO), 50 mg/kg, 100 mg/kg, 150 mg/kg, and 200 mg/kg. Similar doses were measured in the liver [C]. Values are the standard error of the mean fold change normalized to the male parenchyma region of the CO group in the lungs, and male CO in the liver; Rpl13a is the housekeeping gene. Statistical analysis by 2-way ANOVA. *, p ≤ 0.05; **, p ≤ 0.01. Abbreviations: NA, naphthalene; CO, corn oil.

The gene expression of SLC22A4, the ET transporter, in the proximal (Fig. 8A) and distal (Fig. 8B) airway significantly increased in both males (p ≤ 0.05) and females (p ≤ 0.01) exposed to 200 mg/kg of NA when compared to 0 mg/kg. The female liver had maximal expression of SLC22A4 at 50 mg/kg of NA and had significantly lower (p ≤ 0.05) expression at 150 mg/kg of NA compared to 50 mg/kg (Fig. 8C).

### 3.8. Gene expression of CCSP, SLC22A4, GSR, and GPx in female ET pretreated mouse lung

**Fig. 9.**
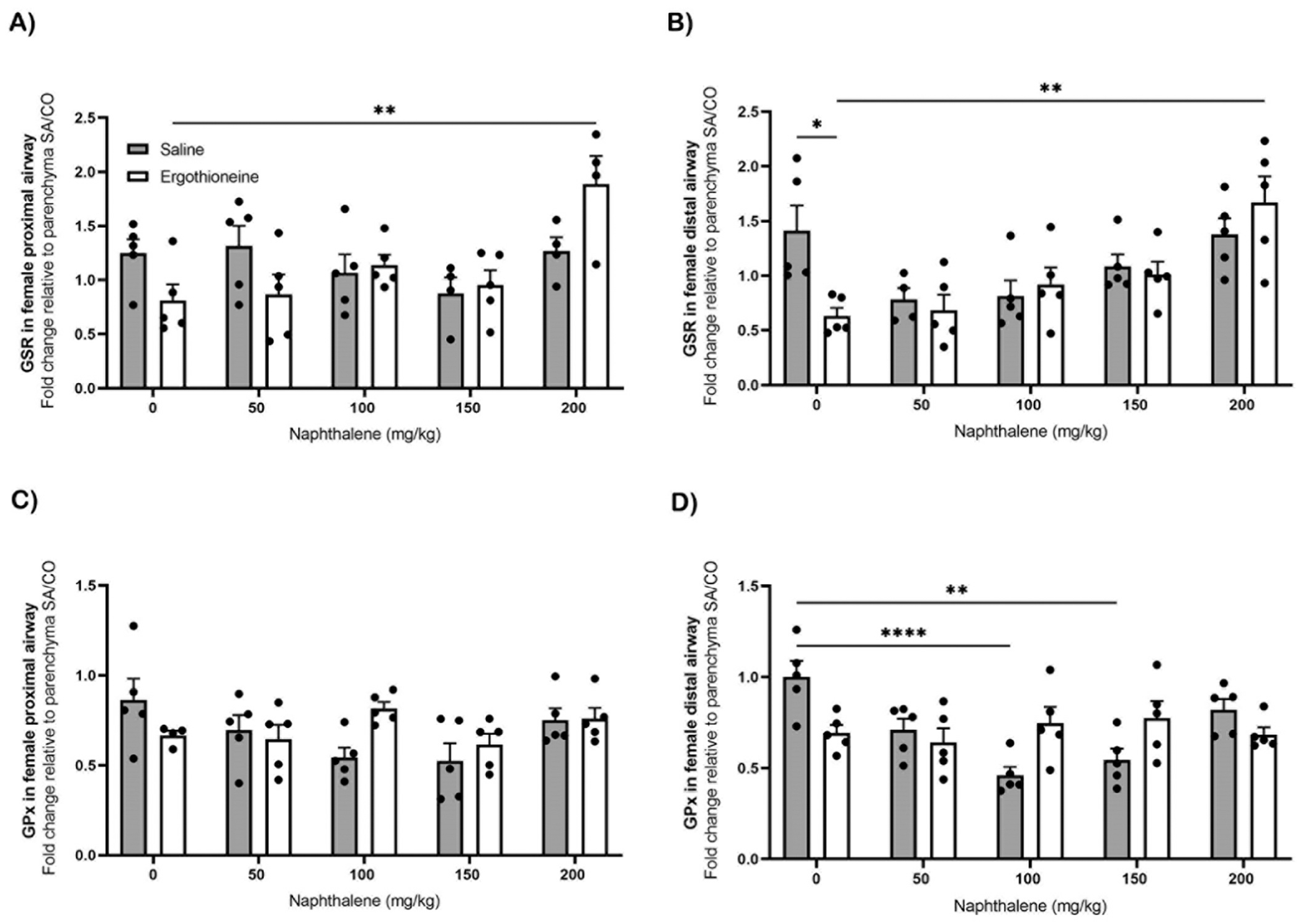
Airway gene expression post ET pretreatment and NA exposure in female mice. GSR proximal [A] and distal [B] airways, and GPx proximal [C] and distal airways [D] were determined by RT-PCR in relation to Rpl13a as the housekeeping gene. Female middle-aged mice, 1–1.5 years of age, were treated with 70 mg/kg of ET for five consecutive days prior to a 24-hour exposure to a NA dose response via ip. Values are standard error of the mean fold change normalized to the parenchyma region of the SA/CO group (n=4–5). Statistical analysis by 2-way ANOVA. **, p ≤ 0.01; ****, p ≤ 0.0001. Abbreviations: ET, ergothioneine; NA, naphthalene; SA, saline; CO, corn oil.

The gene expression in female middle-aged mice was measured to determine if ET pretreatment had any impact on the lung post NA exposure. The gene expression of Club cell secretory proteins (CCSP) (Fig.S6A, S6B), ergothioneine transporter (SLC22A4) (Fig.S6C, S6D), glutathione S reductase (GSR) (Figs. 9A, 9B), and glutathione peroxidase (GPx) (Figs. 9C, 9D) were measured in both the proximal and distal airway of the lung. Female mice treated with SA and exposed to NA had significantly less (p ≤ 0.001) CCSP in the proximal and distal airway as the NA concentration increases (Fig. S6A and S6B). When treated with ET there was significantly less CCSP at 150 mg/kg (p ≤ 0.05) and 200 mg/kg (p ≤ 0.0001) of NA in the proximal airway compared to 0 mg/kg NA (Fig. S6A). Similarly, mice treated with saline in the distal airway had significantly lower expression of CCSP at 100 mg/kg (p ≤ 0.001), 150 mg/kg (p ≤ 0.001), and 200 mg/kg (p ≤ 0.001) compared to 0 mg/kg of NA (Fig. S6B). Mice treated with ET in the distal airway had significantly less expression of CCSP (p ≤ 0.0001) by 2-fold as the NA concentration increased (Fig. S6B).

SLC22A4 expression in untreated female proximal airway was significantly greater in animals exposed to 200 mg/kg NA compared to 100 mg/kg NA. (Fig. S6C). SLC22A4 in the ET treated group had a significantly greater (p ≤ 0.01) expression at 200 mg/kg (p ≤ 0.0001) when compared to 0 mg/kg, 50 mg/kg, 100 mg/kg, and 150 mg/kg (Fig. S6C). When comparing SLC22A4 expression at 200 mg/kg NA in the saline and ET treated group, the mice treated with ET were significantly greater (p ≤ 0.01) in expression than those treated with saline (Fig. S6C). The saline treated group for the distal airway had the greatest expression of SLC22A4 at 200 mg/kg NA (Fig. S6D). The expression of SLC22A4 in the distal airway at 200 mg/kg from mice treated with saline was significantly greater than 100 mg/kg of NA (p ≤ 0.01) (Fig. S6D). When compared to 200 mg/kg NA exposure in mice treated with ET, SLC22A4 was significantly less at 0 mg/kg (p ≤ 0.0001), 50 mg/kg (p ≤ 0.0001), 100 mg/kg (p ≤ 0.001) and 150 mg/kg (p ≤ 0.05) in the distal airway (Fig. S6D).

The expression of GSR in the proximal airway of female mice treated with saline had no significant differences in expression post NA exposure (Fig. 9A). However, when compared to ET treated mice exposed to NA at 200 mg/kg there was significantly less expression at 0 mg/kg (p ≤ 0.01) of NA (Fig. 9A). The distal airway expression of GSR at 0 mg/kg NA was significantly greater (p ≤ 0.05, 2-fold) in saline-treated mice compared to ET treated mice at 0 mg/kg NA (Fig. 9B). Mice treated with ET and exposed to NA at 200 mg/kg NA had significantly greater expression of GSR in the distal airway compared to 0 mg/kg (p ≤ 0.01) of NA (Fig. 9B). The proximal airway expression of GPx had no significant difference between the saline treated and ET treated groups post NA exposure (Fig. 9C). The distal airway of mice treated with saline had significantly less expression of GPx at 100 mg/kg (p ≤ 0.0001) and 150 mg/kg (p ≤ 0.01) compared to 0 mg/kg of NA (Fig. 9D). Mice treated with ET in the distal airway had no significant difference post NA exposure in the expression of GPx (Fig. 9D).

## 4. Discussion

This study is the first to evaluate lung region specific expression of key genes involved in detoxification in aging mice. Further it is the first to conduct a NA dose response in middle-aged mice to define toxicity in the epithelium and evaluate the ability of a dietary antioxidant, ergothioneine (ET), to ameliorate oxidative stress and airway epithelial toxicity. Prior studies have only examined mice up to the age of young adulthood (Plopper et al. 2001; Van Winkle et al. 1995). The regional impacts of NA toxicity in the lung were compared using microdissected lung regions as done in historic and recent studies (Royce et al. 1996; Sutherland et al. 2012). This is especially important when measuring toxicity and cellular impacts in the lung because the response and the dose delivered can vary by location, sex, and age. Surprisingly we found that GSR was less abundant in the lung of females than of males in all regions in middle-aged mice, and this was due to a large increase in GSR expression in middle-aged male mice compared to young adult controls and middle-aged females. We observed accumulation of ET in the lung, liver, and blood, demonstrating absorption and distribution of ET to these organs in middle-aged mice, and that mice not treated with ET, while maintained on an ET free diet, lacked measurable ET. Importantly we were able to show spatial differences in lung ET transporter expression, where the transporter was expressed in both proximal and distal airways but had the greatest expression in the proximal airways. ET pretreatment was able to reduce toxicity in the airways measured as the abundance of vacuolated cells in aged male and female mouse terminal bronchioles, showing some efficacy against NA induced oxidative stress in the lungs.

GCL is the rate limiting enzyme in GSH synthesis and is the key intrinsic intracellular antioxidant in lung cells (Lu, 2009). Normal expression of GCLM in adult mice varies by sex with males having a greater expression compared to females in the intrapulmonary conducting airways, airway bifurcations, and terminal bronchioles (Sutherland et al. 2012). Our study has shown similar results in aging mice, with GCLM having a greater expression in males compared to females in both the proximal and distal airway. In GCLM knock out mice, the lung, liver, pancreas, and other cell types had a significant decrease in GSH expression compared to wildtype (Yang et al. 2002). Previous studies in GCLC knockout mice shared similar results (Dalton et al. 2000). Conversely, overexpression of GCLM and GCLC results in an increased production of GSH (Orellana et al. 2017). A limiting factor in our current study is that we did not examine the protein or enzymatic activity of GCL. However, a previous rat study found that GCL activity declines with aging, although, paradoxically, protein expression of GCLM and GCLC and Nrf2, a biomarker for oxidative stress, increased (Suh et al. 2004). As the oxidative stress in our study increased, we also noted increased gene expression of GCLM and GCLC. The addition of ergothioneine seems to stabilize the expression of both GLCM and GCLC. Overall, these findings support the involvement of GCL in the response to oxidative stress.

Glutathione reductase (GSR) is essential in the detoxification process, with the primary function to reduce oxidized GSSG, to GSH, while glutathione peroxidase will oxidize GSH to GSSG (Zalachoras et al. 2020). In this study we found lower baseline gene expression of GSR in the airways and parenchyma of middle-aged females compared to males in the control group. In addition, we observed middle-aged males have greater expression of GSR compared to adult males in all lung regions. This suggests that middle-aged males potentially have a greater capacity to detoxify oxidative stress through regeneration of GSH compared to adult males or middle-aged females. The mechanism behind the increased GSR in middle-aged males could be the result of aging. Alternatively, this could highlight the peak age at which defense against xenobiotics are most effective in mice. A future direction would be to observe gene expression of GSR in geriatric mice models, roughly 2 years of age and older, in comparison to middle-aged and adults, to fully understand the dynamic expression over the lifespan. GSR increased in expression at 200 mg/kg NA dosing in middle-aged female mice that were treated with ET, supporting previous findings on the relationship between ET and GSR in the detoxification process. Prior studies have shown that GSR is capable of reducing ET as it does for GSH (Jenny et al. 2022). When oxidative stress was induced in adult male rats, endogenous GSH, GSSG, and GSR increased in the airways compared to the control group (Chan et al. 2013). The lungs of GSR knockout mice, a1Neu, are still capable of reducing GSH by stimulating oxidation of NADPH , which is the alternative route for GSH generation in species lacking functional GSR. Sex differences in this route have not been explored. Although functional, GSR knockout mouse was not as effective in the GSH mediated detoxification cycle compared to wildtype mice (Tipple et al. 2007). So, a lower level of GSR such as what was observed for middle-aged female mice in this study would diminish regeneration of reduced antioxidant ET as well as GSH. This is quite concerning and might underpin an elevated susceptibility to oxidative stress, although ability to increase GSR was preserved at high dose challenge with NA but not at lower doses. We did examine gene expression at 24 hrs so it is also possible that the response to NA occurred earlier. However, the baseline level of expression are not likely affected by sham exposure and thus are not likely timing dependent. This may be an aging related phenomenon. Previous studies have shown that ET can directly impact the GPx expression (Liu et al. 2023). Our study also found that GPx is upregulated in middle-aged mice compared to adults. We demonstrated that ET treatment in middle-aged female mice stabilized the production of GPx in the presence of oxidative stress. Thus, ET may play a crucial role in relation to GSH and the detoxification cycle, however more research still needs to be done to understand its impact.

NA induced tissue injury and repair is characterized by a sequence of events that involve GSH loss, vacuolation of injured cells, followed by exfoliation of the injured cells into the lumen of the airway and squamation of remaining epithelia to cover the basement membrane (Van Winkle et al. 1995). Following NA exposure, we observed a depletion of CCSP, an indicator for Club cell abundance by location, as well as histological and stereological indices of NA toxicity in the airways of animals treated with NA. However, based on stereological level of vacuolated cells detected it appears that toxicity was not different by sex in proximal airways and was greater in middle-aged males than in females in the terminal bronchiole. There may be a number of contributing factors to this phenomenon, including: 1) timing of sample collection being 24 hours rather than an earlier time point, 2) vacuolated cells detached from the lumen at the 24 hour time point, 3) the impact of age on the endogenous antioxidants which may impact the strength of ET treatment.

It has been previously shown that ET transporters are found in organs known to experience high levels of oxidative stress and their expression will increase in the presence of cell damage caused by free radicals (Cheah and Halliwell, 2021). For example, the liver is known for its primary role to metabolize xenobiotics, a fact making it susceptible to oxidative stress, which results in a high expression of ET transporter (Tang et al. 2018). In this study we found that the liver ET transporter, SLC22A4, in male mice had no significant change in expression as the NA dose increased. Conversely, females had the highest expression of SLC22A4 at 50 mg/kg and a significant decline in expression at 150 mg/kg NA. This could be evidence of middle-aged male mice metabolizing NA faster in the liver compared to females. Other known impacts of ET pretreatment includes reduced risk of stroke in rodent models (Ong et al. 2022) and reduced risk of the development of mild cognitive disorders in middle-aged humans (Wu et al. 2022). ET has been suggested to be a potential treatment for Coronavirus patients (Cheah and Halliwell, 2020). Overall, scientists have shown that ET has potential in improving the health of both animal and human models; yet there is still more research to be done to truly understand the full functional capability of ET. Our findings emphasize the potential for ET to ameliorate toxicity by NA exposure in middle-aged male mice.

In regard to studying middle-aged mice, this study is a first to demonstrate sex differences in the lungs, understand NA toxicity, and explore the impacts of ET on GSH synthesis in both the liver and lungs. This is a murine correlate of a large US demographic age group, the baby boomers born 1946–1964, who are now progressing from middle age to old age (ages now are 59–75) (Colby and Ortman, 2014). Understanding lung biology during aging, including ages that precede the most elderly age brackets, is going to be important in understanding potential health impacts in these groups. Our findings are the initial step needed to understand lung specific responses in middle-aged mice including key capabilities to detoxify xenobiotics in the lung and the potential benefits of introducing a dietary antioxidant. Key gene expression differences that were found in this study exposed areas of susceptibility by age, sex and location in the lung. Further, this study found that ET was a potential pretreatment for NA toxicity with a modest effect on reduction of toxicity at some doses of NA and at some locations when given orally.

## Supplementary Material

1

## Figures and Tables

**Table 1-:** ThermoFisher Scientific TaqMan Assays used.

Target	Gene aliases	Gene name	Assay ID
RPl13a	tum-antigen	ribosomal protein L13A	mm01612987_g1
Scgb1a1	CCSP, CC10, CC16	secretoglobin, family 1 A, member 1 (uteroglobin)	Mm00442046_m1
Slc22a4	Octn1	solute carrier family 22 (organic cation transporter), member 4	Mm00457739_m1
Gstp1	GstpiB	glutathione S-transferase, pi 1	Mm00496606_m1
Gclm	AI649393, Gcmc, Glclr	glutamate-cysteine ligase, modifier subunit	Mm01324400_m1
Gclc	D9Wsu168e, Glclc	glutamate-cysteine ligase, catalytic subunit	Mm00802655_m1
Gsr	AI325518, Gr-1, Gr1	Glutathione S reductase	Mm00439149_m1
GPx	CGPx, GPx-1, GPx1	glutathione peroxidase 1	Mm04207457_g1

## Data Availability

Data will be made available on request.
