# Supplementary material for: Impact of aging and ergothioneine pre-treatment on naphthalene toxicity in lung
Source: Toxicol Lett. Author manuscript; Available in PMC 2024 Nov 2. (PMC11531314; doi:10.1016/j.toxlet.2024.05.014)

**Supplemental Figure 1- GSH, GSSG and GSH/GSSG** **ratio in the lung.**  Male and female middle-aged mice (n=3-5) have GSH **[A-B]**, GSSG **[C-D]** levels measured using HPLC-MS post ET pretreatment and 24 hours after NA exposure to 50 and 150 mg/ kg i.p. GSH/GSSG ratio, an indicator of lung health, was also evaluated in both males **[E]** and females **[F]**. Statistical analysis by 2-way ANOVA. *, p ≤ 0.05; **, p ≤ 0.01; ***, p ≤ 0.001; ****, p ≤ 0.0001. Abbreviations: ET, ergothioneine; NA, naphthalene

**
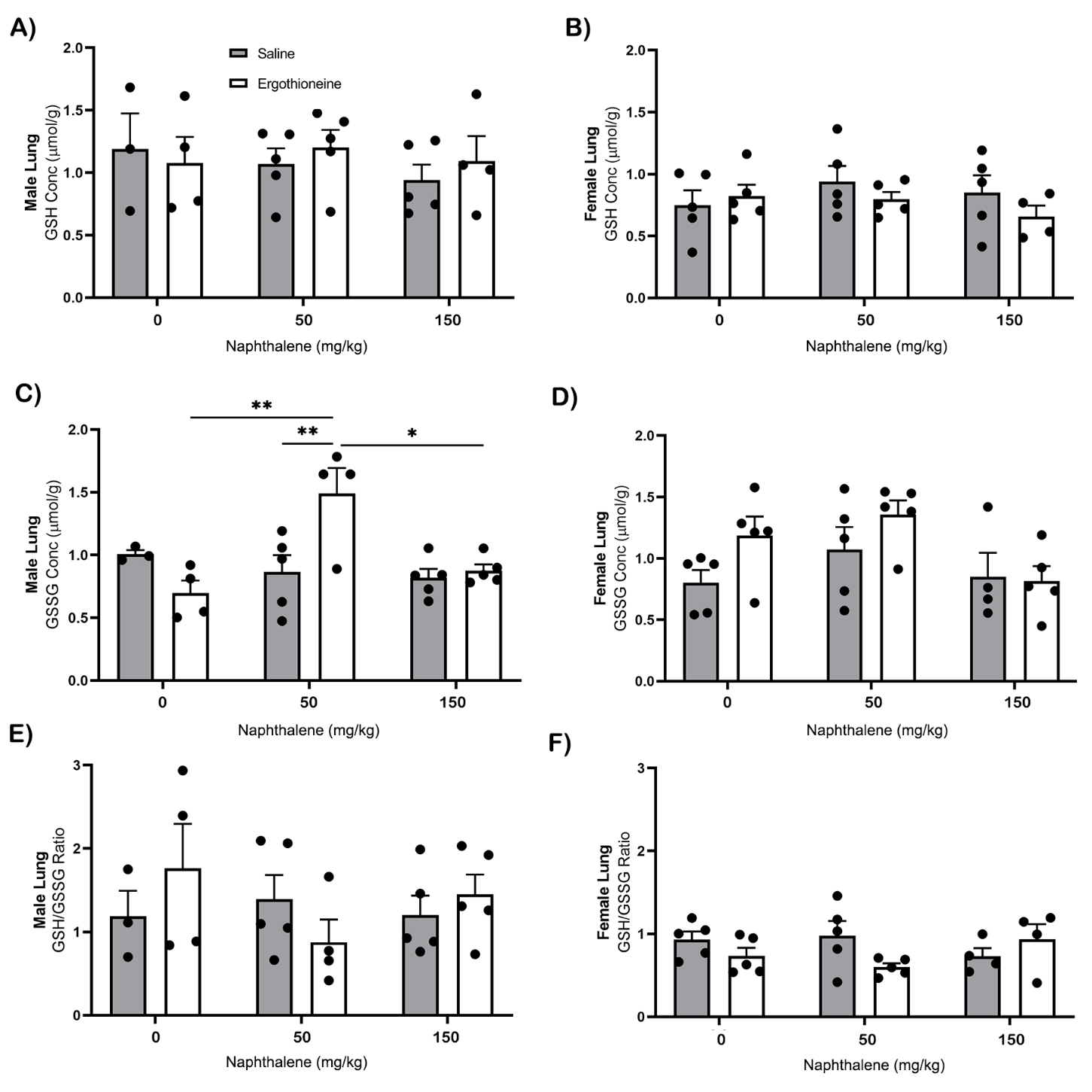
**


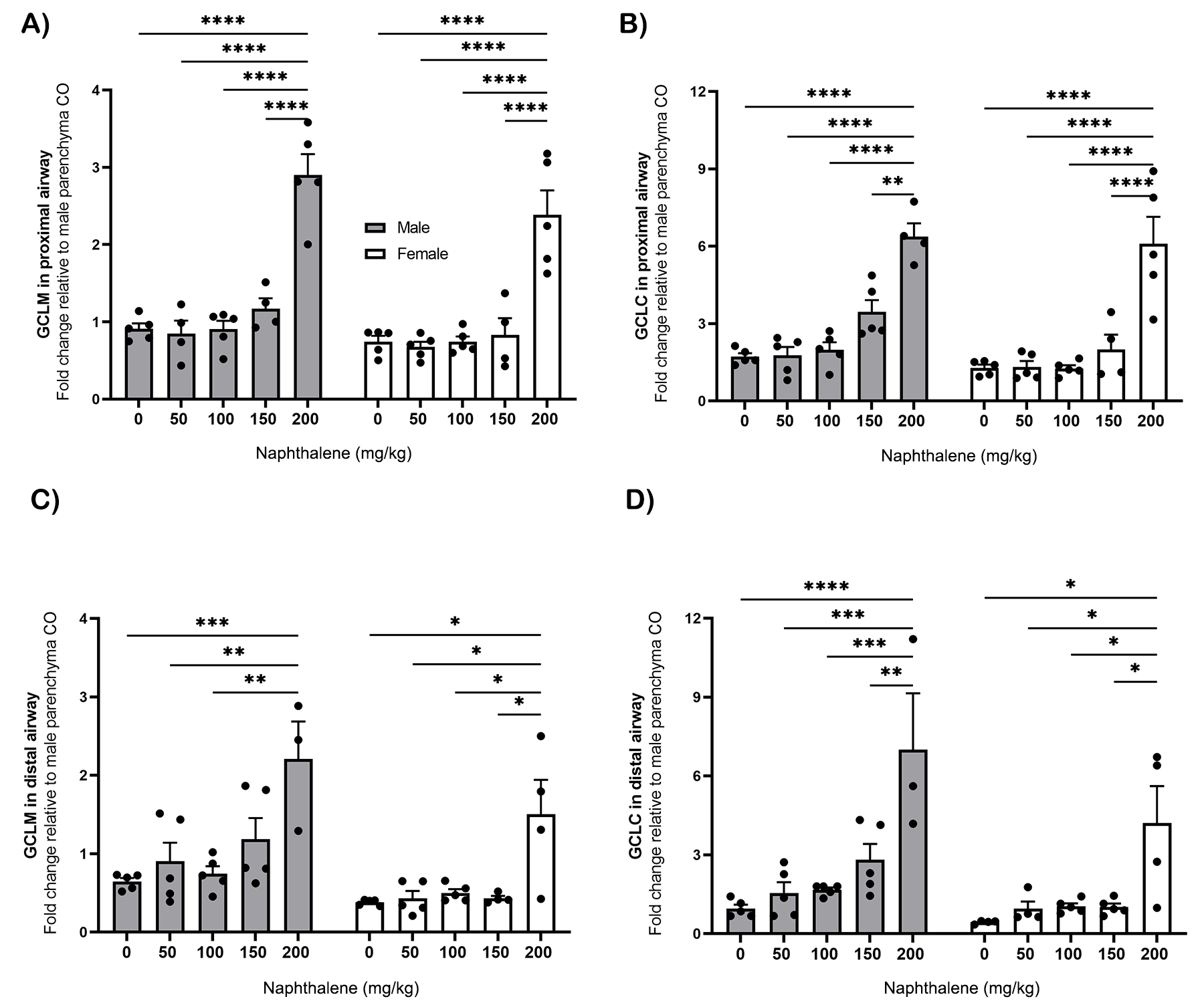
**Supplemental Figure 2- Gene expression related to GSH production in microdissected airways from NA exposed mice.** Microdissected lungs samples from male and female middle-aged mice (n= 4-5) by qRT-PCR. The parenchyma, a lung region containing zero airways, was also collected. Both the proximal **[A and B]** and distal **[C and D]** airways of the lungs were analyzed for GCLM and GCLC in a NA dose response of 0 mg/kg (CO), 50 mg/kg, 100 mg/kg, 150 mg/kg, and 200 mg/kg. Values are the standard error of the mean fold change normalized to the male parenchyma region of the CO group in the lungs with Rpl13a as the housekeeping gene. Statistical analysis by 2-way ANOVA. *, p ≤ 0.05; **, p ≤ 0.01; ***, p ≤ 0.001; ****, p ≤ 0.0001. Abbreviations: NA, naphthalene; CO, corn oil.

**Supplemental Figure 3- Gene expression related to GSH production and detoxification in liver.**

**
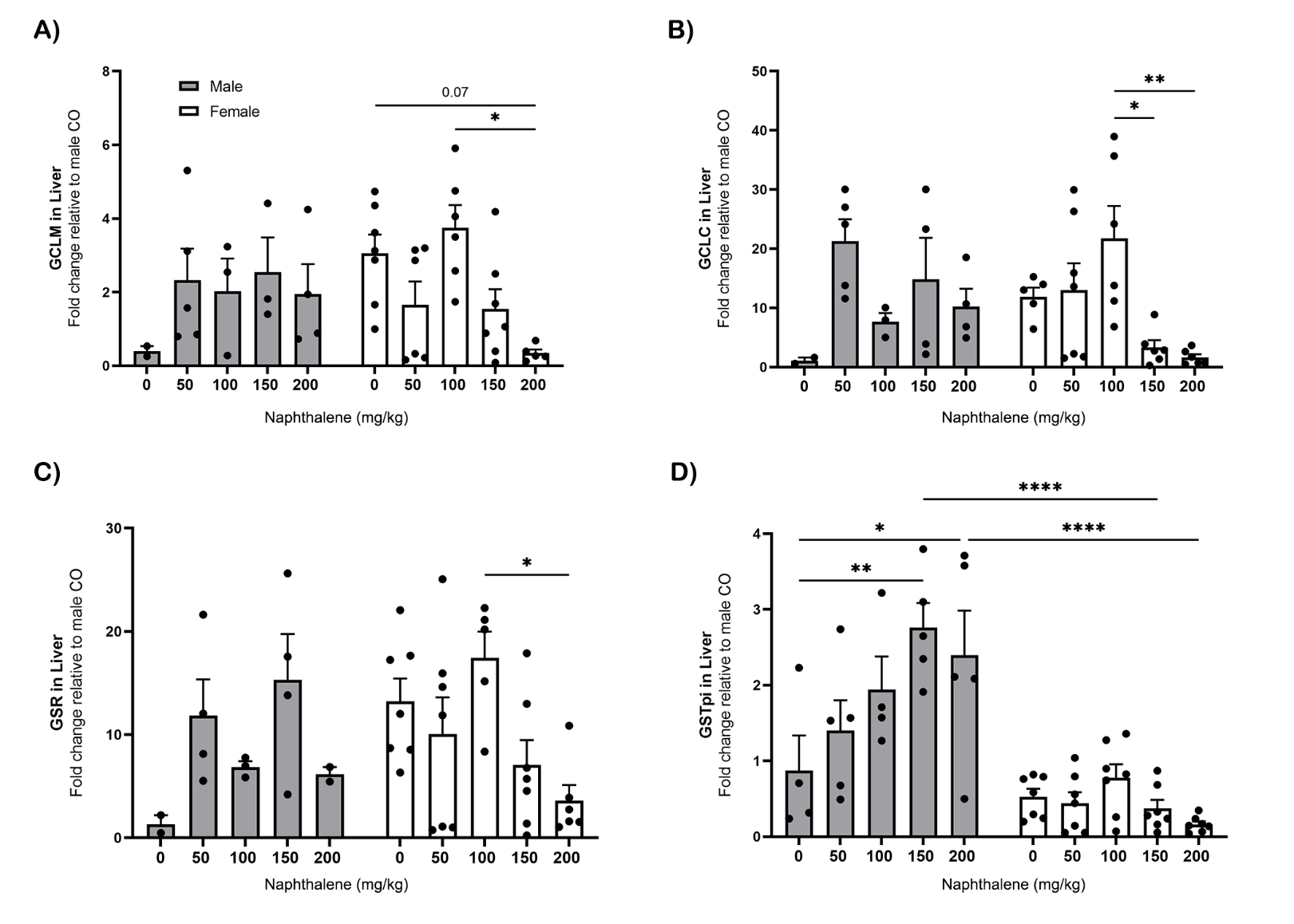
**Liver samples were collected from middle-aged mice (n= 2-6) exposed to 0 mg/kg (CO), 50 mg/kg, 100 mg/kg, 150 mg/kg, and 200 mg/kg of NA for 24 hours. GCLM **[A]**, GCLC **[B]**, GSR **[C]**, and GSTpi **[D]** values are the standard error of the mean fold change normalized to the male CO; Rpl13a as the housekeeping gene. Statistical analysis by 2-way ANOVA. *, p ≤ 0.05; **, p ≤ 0.01; ***, p ≤ 0.001; ****, p ≤ 0.0001. Abbreviations: NA, naphthalene; CO, corn oil.

**Supplemental Figure 4- CCSP gene expression in sham and NA exposed microdissected lungs.** CCSP (Club cell secretory protein) was measured in microdissected lung samples from untreated and NA exposed middle-aged mice (n=5) by qRT-PCR. Both the proximal **[A]** and distal **[B]** airways of the lungs were analyzed in a NA dose response of 0 mg/kg (CO), 50 mg/kg, 100 mg/kg, 150 mg/kg, and 200 mg/kg. Values are the standard error of the mean fold change normalized to the male parenchyma region of the CO group; Rpl13a is the housekeeping gene. Statistical analysis by 2-way ANOVA for normally distributed data sets, and non-parametric Kruskal-Wallis test for datasets not normally distributed. *, p ≤ 0.05; **, p ≤ 0.01. Abbreviations: NA, naphthalene; CO, corn oil.

**
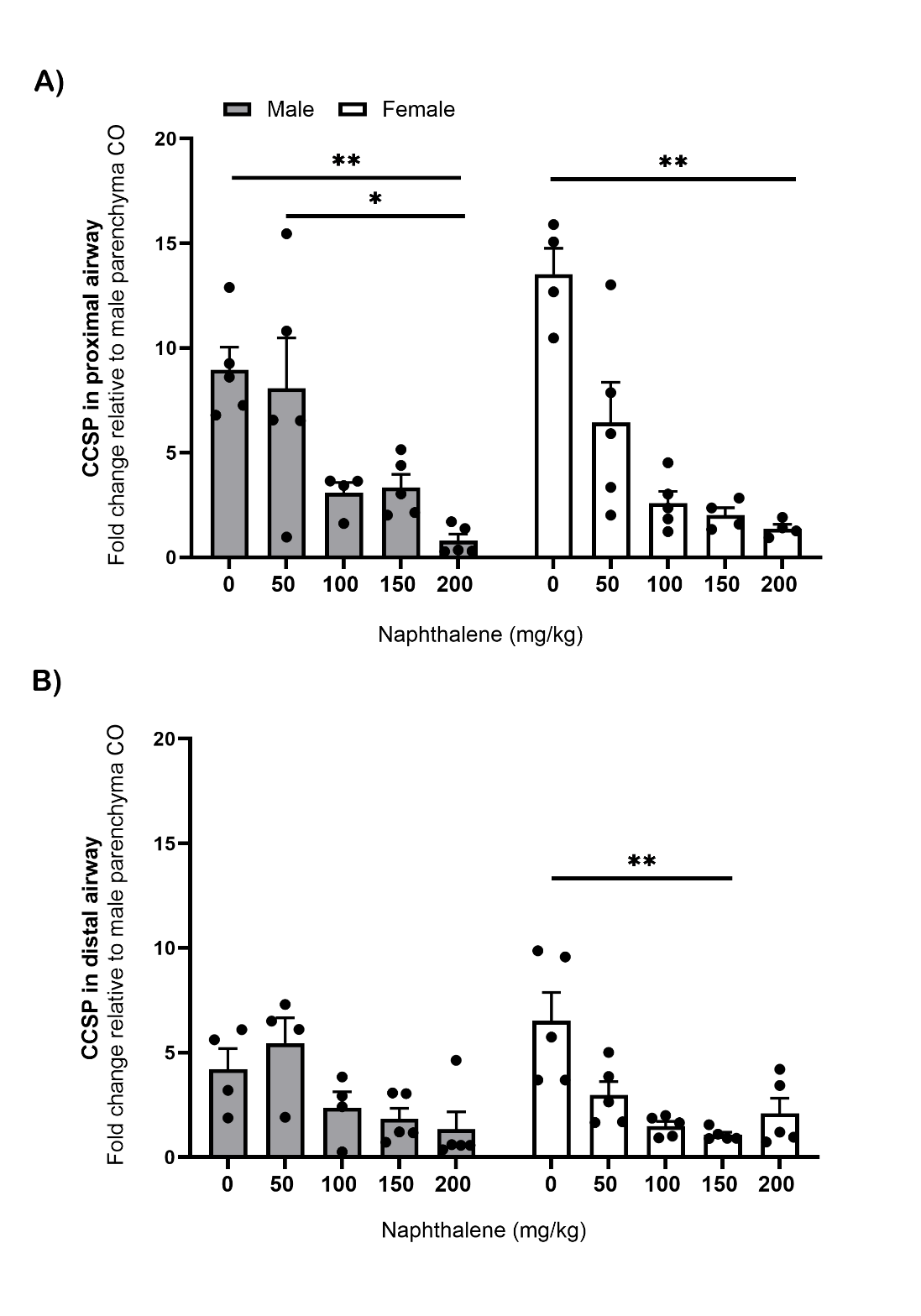
**

**Supplemental Figure 5- Histology and Stereology of ET treated middle-aged proximal airway exposed to 100 mg/kg NA.** Male and female middle-aged mice (n=5) were treated with ET prior to a 24-hour exposure period to 100 mg/kg of NA ip. The airway and terminal airways were imaged using a high-resolution light microscope at 20x, then converted to grayscale [A]. Stereology was conducted using the imaged lungs and the vacuolated cells **[B]**, non-vacuolated cells **[C]** were calculated (n= 4-5). Error bars represent standard error. *, p ≤ 0.05; **, p ≤ 0.01; ***, p ≤ 0.001. Abbreviations: SA, saline; ET, ergothioneine; NA, naphthalene; CO, corn oil.


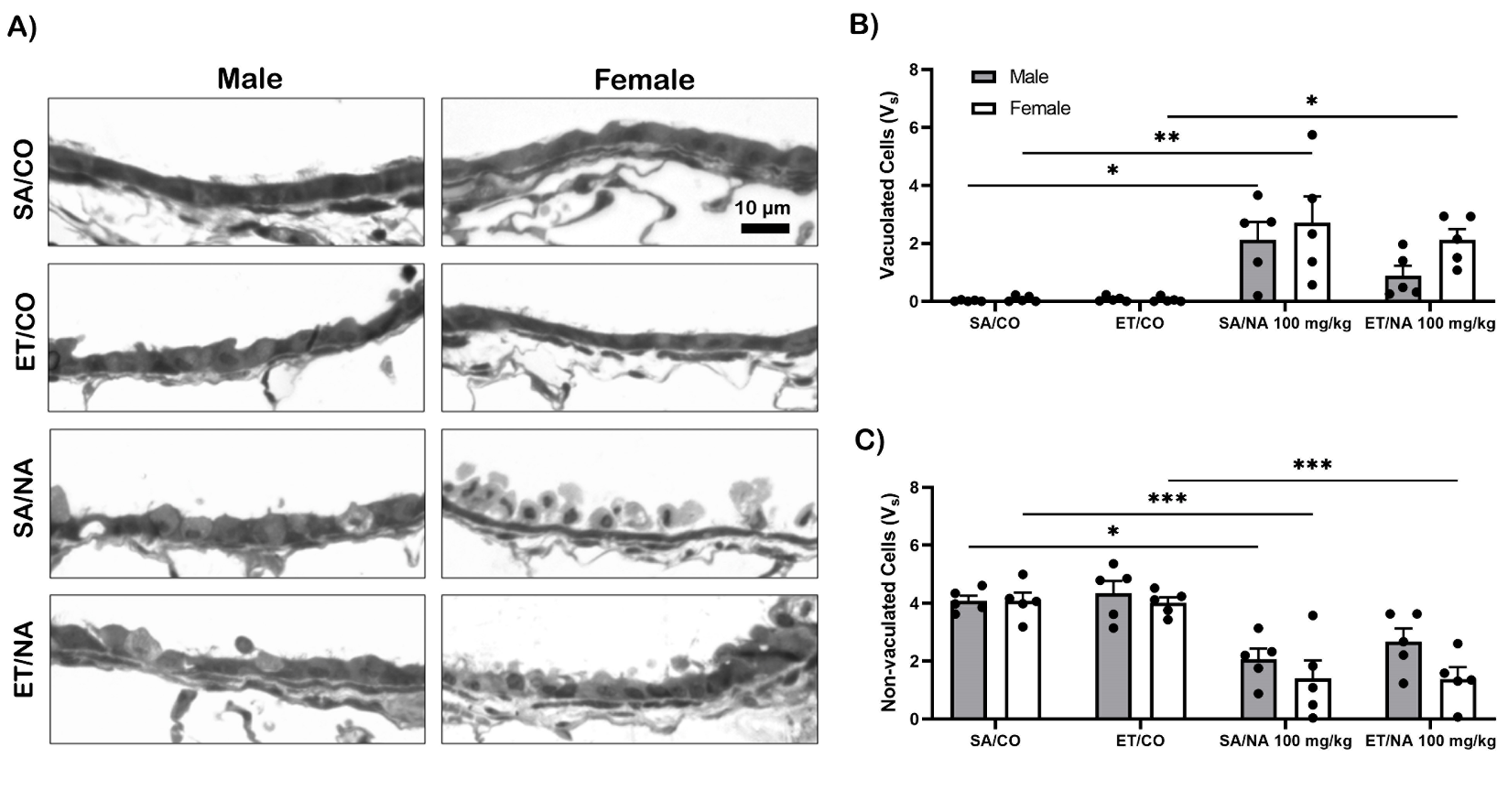


**Supplemental Figure 6- Female gene expression of CCSP and SLC22A4 post ET pretreatment and NA exposure.** CCSP proximal **[A]** and distal **[B]** airways, and SLC22A4 proximal **[C]** and distal airways **[D]** were determined by RT-PCR in relation to Rpl13a as the housekeeping gene. Female middle-aged mice, 1 to 1.5 years of age, were treated with 70 mg/kg of ET for five consecutive days prior to a 24-hour exposure to a NA dose response via ip. Values are standard error of the mean fold change normalized to the parenchyma region of the SA/CO group (n=4-5). Statistical analysis by 2-way ANOVA for normally distributed data sets, and non-parametric Kruskal-Wallis test for datasets not normally distributed. *, p ≤ 0.05; **, p ≤ 0.01; ***, p ≤ 0.001; ****, p ≤ 0.0001. Abbreviations: ET, ergothioneine; NA, naphthalene; SA, saline; CO, corn oil.


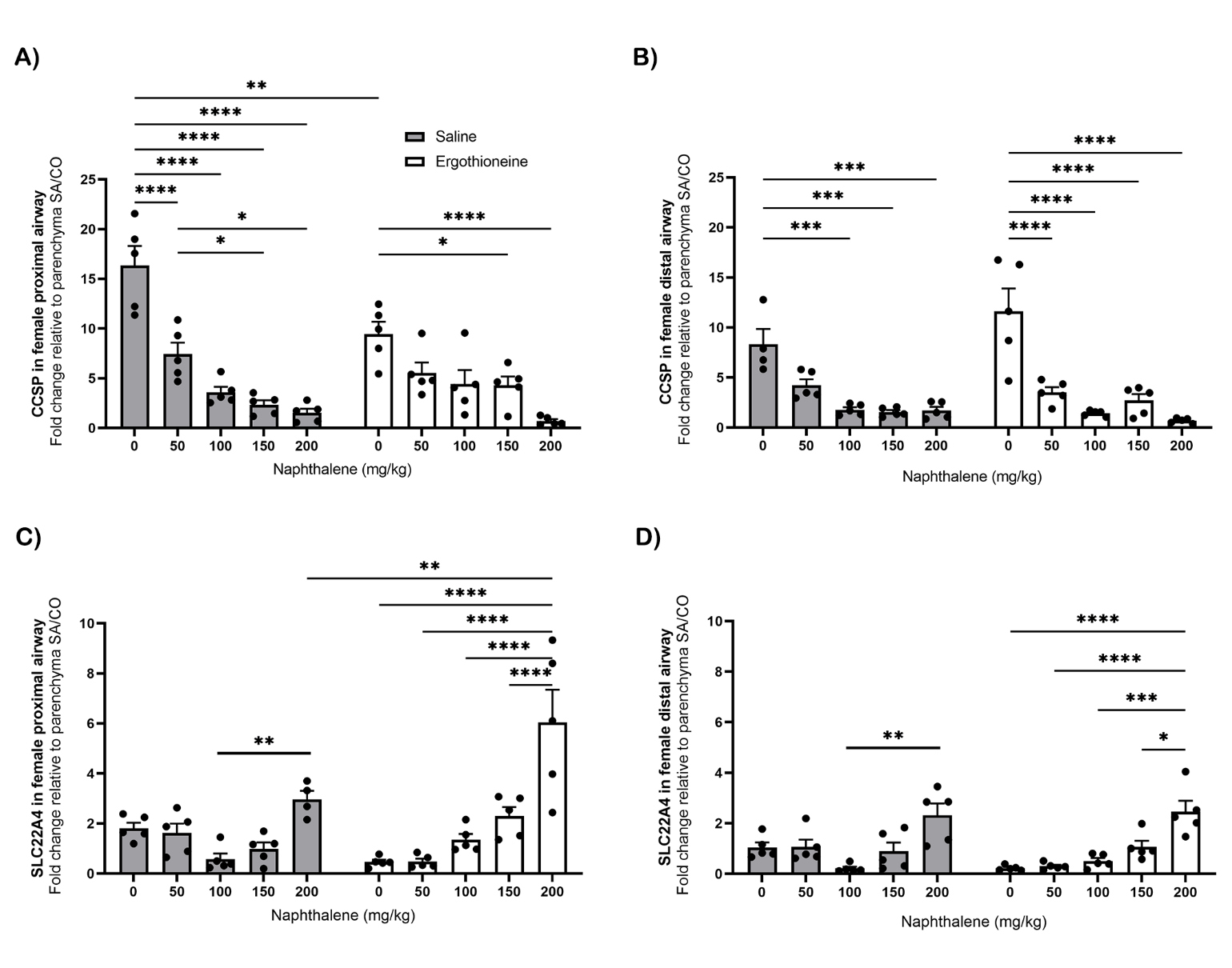

Supplement: 1 [file NIHMS2029986-supplement-1.docx]
